# Qayapu in the reconfiguration of Andean healing and the ritual recovery of the child's soul

**DOI:** 10.3389/fsoc.2026.1870310

**Published:** 2026-10-20

**Authors:** Edgar Gutiérrez-Gómez, Ana Rocío Saboya-Pineda, Rubén Darío Alania-Contreras, Daniela Isabel Dayan Ortega-Révolo, Wilfredo Bazán-Ramírez, Aldo Bazán-Ramírez

**Affiliations:** 1 National Autonomous University of Huanta, Ayacucho, Peru; 2 National University of Central Peru: Huancayo, Junín, Peru; 3 Continental University, Huancayo, Junín, Peru; 4 Universidad Nacional Mayor de San Marcos, Lima, Peru; 5 José María Arguedas National University, Andahuaylas, Apurímac, Peru

**Keywords:** Andean healing, calling of the soul, fright, *mancharisqa*, soul

## Abstract

The research analyzes Qayapu (calling of the soul) as a current practice in the reconfiguration of Andean healing, associated with the ritual recovery of the child's soul (*mancharisqa*), in tension with biomedical medicine. Its persistence is examined in urban spaces of Ayacucho, where differentiated therapeutic systems coexist. The study adopts a qualitative ethnographic approach, grounded in medical anthropology and hermeneutic interpretation. Participant observation, in-depth and semi-structured interviews in Quechua and Spanish, life histories, and field diary records were employed, using intentional and snowball sampling. Data analysis was conducted through inductive thematic coding and categorization. The findings indicate that *susto* is interpreted as soul loss, with physical and emotional manifestations that guide the use of Qayapu as treatment. The ritual, of a syncretic nature, is performed in public spaces and demonstrates symbolic efficacy, reflected in patients' relaxation and drowsiness. Distrust toward biomedicine and restricted access to specialists are also identified. It is concluded that Qayapu persists as an active therapeutic system, whose understanding requires intercultural approaches that integrate ancestral knowledge with mental healthcare.

## 1 Introduction

In the Peruvian Andes, childhood illness and wellbeing are understood through culturally specific systems of knowledge that coexist with biomedical and psychological approaches. One such condition is *mancharisqa* (the frightened person), an emic category associated with fright or intense fear and understood through the belief that the soul may become separated from the body as a result of a frightening experience or other external circumstances. Its treatment is carried out through the Qayapu ritual, or “calling back the soul,” which seeks to restore the relationship between body and soul according to Andean understandings of illness and healing. Although Qayapu has its origins in rural Andean communities, the practice has also moved into urban spaces, where it remains active and families continue to seek ritual specialists for their children. Despite the significant body of research on ancestral knowledge in the Peruvian Andes, Qayapu has received limited attention as an emic category with substantive significance for understanding childhood and adult illness, healing, and wellbeing. Its continuity in urban settings—including city centers and daytime spaces, in contrast to its more private and often nighttime practice in rural Andean communities—also reveals processes of adaptation and transformation while preserving its cultural significance.

Qayapu, understood in the Andean tradition as the “calling back of the soul,” is a ritual practice associated with the healing of individuals affected by fright or intense fear. According to this belief system, a frightening or emotionally distressing experience may cause the soul to temporarily leave the body or become held by entities of the sacred landscape, such as hills or mountains. The resulting condition is understood through a culturally specific relationship between body and soul that persists until this relationship is restored. The ritual specialist performs a series of symbolic actions intended to call and guide the soul back to the patient's body, a process that constitutes the core of healing within this Andean tradition. This practice is especially common among children, who are considered more vulnerable to soul loss following experiences of fear or sudden fright. From the participants' perspective, the therapeutic significance of Qayapu lies in restoring the relationship between body and soul and in addressing the condition associated with *mancharisqa*. Rather than evaluating physiological or clinical effects, the study examines how healing and therapeutic efficacy are understood and experienced by participants within a cultural conception of the soul and its relationship to the body.

The practice of Qayapu has gained relevance in urban settings through rural-to-urban migration processes, in which kinship and reciprocity networks continue to play a fundamental role in organizing social life. In the Andean system, “the strengthening of social ties involving reciprocal obligations becomes imperative. In the rural Andean system, kinship relations entail a series of mutual social obligations and constitute the fundamental fabric of the social structure” (Chagnollaud, 2013, p. 449). This relational structure facilitates the intergenerational transmission of knowledge, ritual practices, and ways of understanding life, illness, and death. In this regard, “the existential dimension is articulated through orally transmitted rituals and beliefs that express conceptions of life and death. These beliefs are communicated primarily in Quechua, an ancestral language still spoken in several regions” (Gutiérrez-Gómez et al., 2025, p. 2). Consequently, Qayapu is embedded within a knowledge system that connects conceptions of health, spirituality, and community cohesion. As Ramos Lopez et al. (2025) note, “its role in healthcare is fundamental, especially in rural communities and urban sectors where access to biomedicine is limited or where people rely on this knowledge as part of their worldview” (p. 3). Thus, even in urban contexts where modern medicine predominates, Qayapu remains a socially legitimate and culturally significant practice through which families and practitioners understand and address experiences of illness and healing.

From this perspective, *Qayapu* can be understood as a therapeutic practice of traditional Andean medicine associated with the restoration of the soul, whose persistence in urban settings does not represent a mere reproduction of the past but rather a process of cultural adaptation and re-signification. Migration has not led to the abandonment of this ritual knowledge; instead, it has been reconfigured within new social environments while preserving the symbolic principles that connect body, soul, and community. This process can be interpreted through the concept of cultural hybridity proposed by García Canclini (1989), according to which traditional cultural practices interact with new social and cultural contexts, generating renewed forms of expression without completely losing their fundamental meanings. Consequently, *Qayapu* illustrates the capacity of Andean systems of knowledge and healing to adapt to urban environments while preserving the cultural meanings and therapeutic significance attributed to the ritual by practitioners and families, as well as its enduring relevance as a living cultural heritage.

From the perspective of habitus theory, these practices can be understood as socially acquired and internalized dispositions that guide action and contribute to the continuity of cultural practices across different social fields. In this regard, “While Bourdieu offers a general view of habitus within the dominant pattern of schooling, when Bernstein's pedagogical discourse is applied, habitus can be observed much more explicitly and how it permeates experiences classified through the hierarchy of symbolic power” (Niati, 2018, p. 2). Within this perspective, Qayapu can be understood as an embodied and socially learned practice whose reproduction extends beyond its original rural setting into urban spaces while preserving shared cultural meanings. Cultural circulation further helps explain the mobility and transformation of these practices within broader processes of social change. “A central concern of these works is the dynamic and disjunctive nature of global cultural flows and the transformative impact of globalization on imagination and identity, including its contradictions and potential for conflict” (Luong Thi, 2025, p. 158). From this perspective, the movement of Andean populations also involves the circulation of knowledge, ritual practices, and culturally situated understandings of illness and healing, allowing Qayapu to be reterritorialized and re-signified within new urban social spaces.

*Mancharisqa*—a cultural category associated with experiences of “fright” in childhood—is understood and addressed through Qayapu, or the “calling back of the soul,” whose therapeutic significance is recognized by the social actors involved. This practice is embedded within a cultural system that encompasses beliefs, experiences, and particular ways of understanding childhood illness, wellbeing, and healing. The objective of this study is to analyze Qayapu as an Andean emic therapeutic practice associated with the restoration of the soul in childhood, examining its meanings, social legitimacy, and persistence in urban settings. Rather than assessing the physiological or clinical effects of the ritual, the study seeks to understand how Qayapu, in relation to *mancharisqa*, articulates culturally situated conceptions of the body, soul, illness, and healing and how these meanings are maintained and reconfigured within urban social life.

The contribution of this study lies in examining Qayapu as an emic category that has received limited attention in scientific literature and as a culturally situated therapeutic practice within ancestral Andean medicine. The study contributes to understanding its continuity and adaptation in urban settings, where its social legitimacy and therapeutic significance are recognized by practitioners and users. It also highlights the importance of an anthropological approach to childhood illness and wellbeing, particularly when culturally specific understandings of health and healing differ from dominant biomedical and psychological frameworks. The manifestations associated with *mancharisqa* are recognized and interpreted by Qayapu practitioners within their own system of knowledge, which provides a distinct framework for understanding childhood experiences of fright and illness. In this regard, “the pharmaceutical industry has set the agenda for research on psychiatric medications, seeking to provide evidence that promotes the marketing and positioning of specific drugs of questionable effectiveness” (Kirmayer and Pedersen, 2014, p. 768). This perspective underscores the importance of critically examining dominant approaches to mental health and recognizing that therapeutic knowledge and practices are also shaped by cultural, social, and institutional conditions.

## 2 Materials and methods

This research was conducted within a qualitative ethnographic approach aimed at understanding the meanings, practices, and therapeutic rationalities associated with Qayapu as an Andean healing practice in an urban setting. The study is grounded in medical anthropology and interpretive hermeneutics, focusing on the meanings attributed by practitioners and users to illness, healing, ritual practices, and the body–soul relationship. A descriptive–interpretive scope was adopted to characterize Qayapu practices and examine how the actors involved understand and experience the healing process. The unit of analysis comprised Qayapu healing practices and the narratives and experiences of Qayapu practitioners, adult users, mothers, and other community members involved in or accompanying the therapeutic process. Fieldwork was conducted in public and community spaces in the city of Ayacucho, particularly the atrium of the Church of San Francisco de Asís and the Central Market of Huamanga, where Qayapu practitioners provide their services and where interactions between practitioners, patients, and accompanying family members can be observed.

Fieldwork was conducted between January and May 2026 through participant observation and direct observation in the atrium of the Church of San Francisco de Asís and the Central Market of Huamanga, public spaces where Qayapu is practiced. The fieldwork was carried out by three researchers, all of whom are Quechua- and Spanish-speaking, which facilitated direct communication with participants in Ayacucho Quechua or Spanish according to their preferences. Observations focused on interactions between Qayapu practitioners, patients, and accompanying family members, including people who had migrated from rural Andean communities. Semi-structured and in-depth interviews were conducted primarily at the practitioners' urban workplaces and in community spaces where the rituals take place. Several interviews and ritual encounters were audio-recorded and filmed with prior informed consent, and the recorded material was subsequently transcribed and reviewed alongside the researchers' field notes. Data analysis was conducted manually, without the use of qualitative data analysis software, through an inductive and interpretive process that identified recurrent themes related to *susto, mancharisqa*, the body–soul relationship, symbolic efficacy, syncretism, and therapeutic practices. The researchers reviewed the field material collectively to strengthen interpretive coherence and analytical credibility. Data collection continued until thematic saturation was reached.

In-depth interviews were conducted with nine Qayapu practitioners, including five women and four men. The practitioners reported similar therapeutic procedures and approaches to the healing ritual, although their practices were situated in different urban spaces. The women practiced primarily in the urban area of Huamanga, including the Central Market, where they combined ritual healing with itinerant vending of various products. The four male practitioners received patients in the atrium of the Church of San Francisco de Asís, where they attended to people seeking treatment. Twenty adult patients who sought Qayapu healing were also interviewed. Many of these adults brought their children for treatment and, in some cases, also received healing themselves. Children of both sexes were the group most frequently brought to the practitioners during the fieldwork, particularly in cases locally described as *mancharisqa*. Informal conversations and interviews were also conducted with other city residents encountered during fieldwork. Participants were selected through purposive sampling to identify individuals with direct experience of Qayapu, followed by snowball sampling to recruit additional participants recommended by initial informants. During fieldwork, the practitioners performed the same ritual procedures used with other patients, which enabled direct participant observation of the healing practice (Figure 1). This sampling and observational strategy allowed the study to include practitioners, adult users, mothers and accompanying family members, and cases involving children, thereby capturing variation in the ways Qayapu and *susto* were understood and experienced.

**Figure 1:**
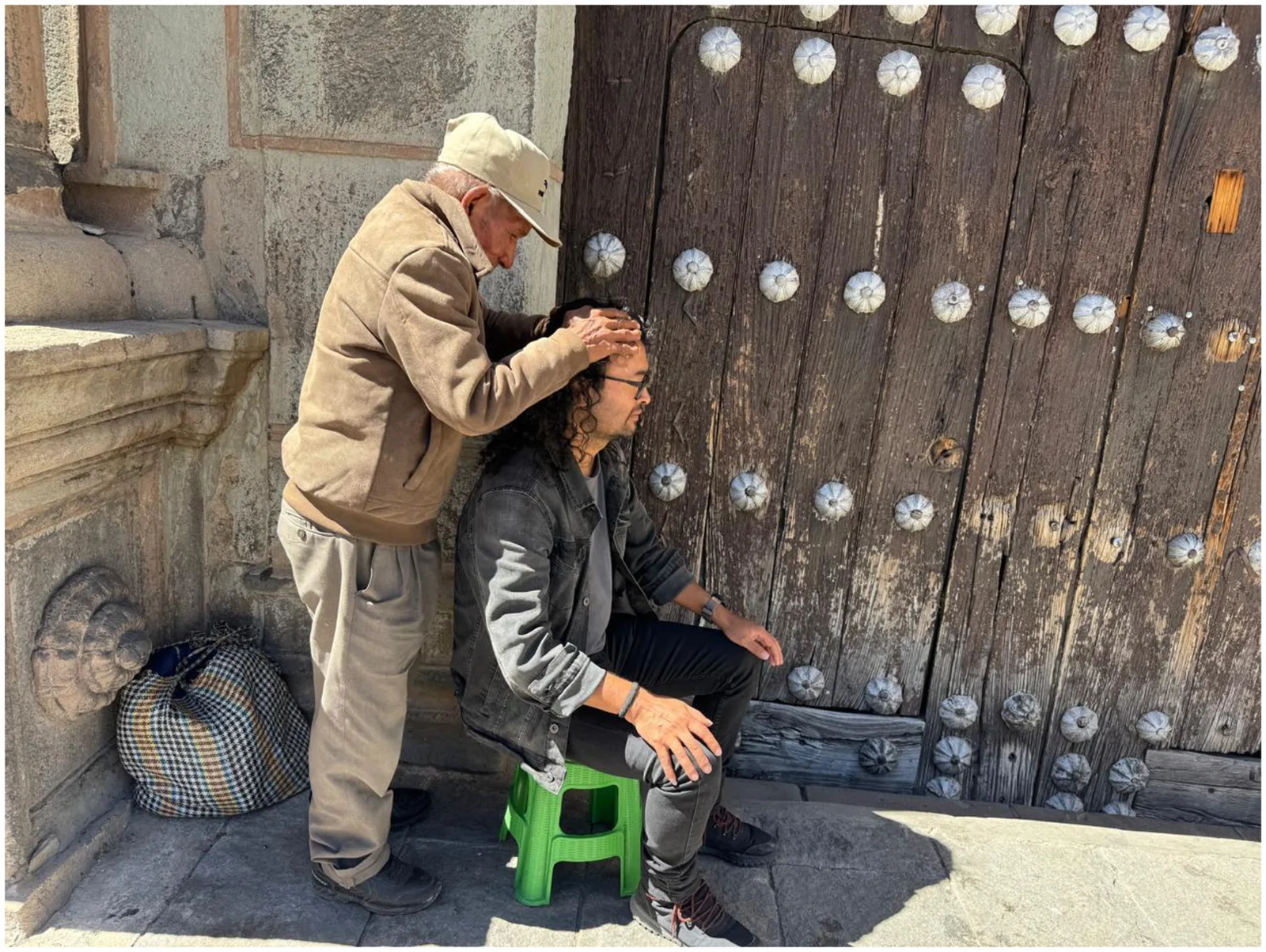
During one of the participant observation sessions, one of the researchers took part in the ritual healing process. During the ritual, the specialist uttered the following expressions in Quechua: “*Maldición, Satanás, lluqsiy chay umamanta, dejay alman kutimunanta, llumpay mancharisqam kachkan,”* while performing a ritual blowing action directed toward the patient's head. According to the specialist's interpretation, these words are intended to expel the evil that has taken possession of the person's brain or mind and to facilitate the return of the soul, which is believed to have separated from the body as a result of intense fright. This act constitutes one of the central moments of the Qayapu therapeutic process. Photograph taken by the authors, May 2026.

Fieldwork was conducted primarily in Ayacucho Quechua, although Spanish was also used according to participants' linguistic preferences and communicative practices. The three researchers' proficiency in both Quechua and Spanish facilitated direct interaction with participants and reduced the need for external interpreters. Participants' statements were documented in their original linguistic form whenever possible, and particular attention was given to preserving their meanings during transcription and translation. This was especially important because some participants used brief expressions and non-standardized combinations of Quechua and Spanish, reflecting diverse forms of bilingualism and local patterns of communication. The translation process therefore prioritized semantic and cultural fidelity rather than literal word-for-word equivalence.

Informed consent was obtained prior to audio recording, video recording, interviews, and researcher participation in ritual procedures. Participant observation and general observation were also conducted in the public and openly accessible spaces where Qayapu rituals routinely take place. In these cases, the researchers did not interfere with therapeutic procedures or participants' decisions, and the observations focused on interactions, ritual practices, and the broader social setting of healing. Particular care was taken to distinguish between information obtained through consented interviews and recordings and observations made in publicly accessible settings.

## 3 Results

### 3.1 The cultural construction of illness as a mental imbalance

Within Andean culture, the soul, as the spiritual dimension of the human being, is conceived in duality with the body, and balance depends on the articulation between the two. Soul imbalance is not understood as an individual event but as the result of external factors from the social and natural environment. In childhood, *mancharisqa* (fright or fear) is understood as an emic category associated with a disruption in the relationship between body and soul. This process is explained by healing specialists, who maintain that it does not involve the definitive loss of the soul but rather a condition that can be reversed through ritual practices: “it is fright that makes the little soul sick; for that reason, we pray so that the person may heal. The soul does not leave, it does not come out of the body; if it was left, we would die. We simply call it so that it may heal. If we do not pray and it is not cured, the person may even become insane.” This perspective reflects a dynamic understanding of the soul, whose stability can be symbolically restored. In this context, Qayapu is based on the idea that the spirit is mobile: “it tends to abandon the body and, with death, to separate permanently and become a soul, which, as I have mentioned, is also the generic form used in the Andes” (Morán, 2022, p. 69). This expresses an Andean ontology in which the spirit is mobile, separable from the body, and becomes a soul upon definitive death.

The precise origin of practices aimed at restoring children's soul balance remains unknown. The term “healer” is considered inadequate from the local perspective, since community members recognize these agents as specialists endowed with a “privileged soul,” culturally legitimized to intervene in healing processes. *Susto* (“fright”) is understood in this study through the local category of *mancharisqa*, which refers to an experience of fright associated with an imbalance in the relationship between body and soul. In this context, it has been noted that “the Quechua shepherds of the Ausangate highlands have developed effective discourses in which the ailment of ‘susto' cannot be resolved without resorting to distinctive forms of exorcism” (Fernández Juárez, 2019, p. 492). These discourses constitute symbolic therapeutic frameworks in which the restoration of soul balance depends on socially legitimized ritual practices.

From the perspective of specialized practice, one ritual specialist stated: “I have to heal them up to three times because it is the devil who causes the fright. After three sessions it passes; the first time they calm down, and by the second and third they already feel better.” This therapeutic sequence reflects a process-oriented understanding of healing in which the repetition of the ritual is regarded by the specialist as part of the healing process. Narratives collected through interviews reveal the wide range of situations associated with the origin of *susto*: “They can be frightened anywhere, but mostly in the fields, from falls, animals, or at night. Those who are frightened in the water become mute; they cannot speak.” These accounts illustrate a culturally constructed etiology in which everyday events are interpreted as triggers of soul imbalance. Within the study setting, *susto* is understood as an explanation for various childhood conditions, and Qayapu is practiced as a culturally meaningful form of healing associated with this experience of fright.

The persistence of *susto* as an etiological category is sustained by recurring practices and oral narratives in Quechua that have been transmitted across generations. According to one participant, its symptoms are manifested as follows: “They usually have a fever, headaches, become pale, vomit, and cannot sleep.” Some mothers reported seeking Qayapu practitioners for their children because they understand *mancharisqa* as a condition that requires a culturally specific form of healing, distinct from biomedical or psychological approaches. Nevertheless, this culturally specific understanding of *mancharisqa* coexists with broader scientific perspectives on childhood fear. As Pérez Grande (2000) notes, “the study of fear and its role in child development have made significant progress in recent years thanks to contributions from various disciplines—including ethology, psychology, education, biology, and pharmacology” (p. 123). In this study, this perspective is not used to assess the physiological or clinical effects of Qayapu, but rather to situate local understandings of childhood fright alongside broader approaches to the study of fear.

The learning and legitimization of these practices occur primarily within the family sphere through the intergenerational transmission of knowledge. As one interviewee explained: “My grandparents and my mother used to tell us that Qayapu happens when a little soul, someone, a spirit, our ancestors, or some presence that was there takes hold of the baby. Since they are pure souls, not with bad intentions, but simply because they see them or for some other reason. For many reasons this happens to children and they become frightened; that is why the Qayapu is performed, so that the soul may return and health may be restored.” Consequently, Qayapu is understood by practitioners and families as a culturally meaningful healing practice for addressing childhood experiences associated with *mancharisqa*, whose relevance is not limited to rural settings but is also maintained and reproduced in urban spaces.

Migration is one of the determining factors that has enabled the presence of the Andean healing system in urban areas, particularly in the Historic Center, the designation given to the area by municipal authorities. The key informant, who carries out this activity daily in the church square, stated that he arrived with his family on the outskirts of the city as a result of the violence perpetrated by the armed movement Shining Path. He explained that he inherited this practice from his parents, who performed it as part of their activities in the countryside. The informant indicated that he learned this vocation as an activity closely linked to rural life. Currently, due to his advanced age, he devotes himself primarily to this work, which he regards as an inherited gift. He noted that none of his children are interested in learning this knowledge and expressed concern that the tradition may eventually disappear. He also reported facing difficulties in carrying out his work because of restrictions imposed by municipal workers and local security personnel, who, in his view, do not allow them to conduct their activities peacefully. Finally, he called on municipal authorities to create spaces for the promotion and appreciation of this cultural practice as an expression of Andean knowledge.

The therapeutic practices of Qayapu vary according to locality, the specialist, and the nature of the cases being treated. The interviews were conducted in an urban context in Ayacucho, where the ritual remains active and is attended by users who bring their children for the treatment of *susto* (fright). One specialist described this diversity in modes of intervention by stating: “Each person has their own way of doing it; it is different. The number of clients has not decreased—they always come looking for me. More people come on Tuesdays and Fridays at exactly noon, although it can be done at any time.” This testimony reveals the existence of therapeutic differentiation, a sustained demand over time, and the presence of specific temporal patterns of care, suggesting a well-established symbolic legitimacy within local community cultural practices. These practices can also be understood in relation to culturally specific conceptions of the relationship between emotions and the body. Within the humoral conception of illness, “intense emotions load the blood with humoral heat or cold; and emotions of fear cool the blood” (Quinlan, 2010, p. 2). In this study, this conception is considered as part of the local explanatory framework surrounding *susto*, rather than as evidence of a physiological effect produced by Qayapu.

### 3.2 A ritual healing practice based on syncretic and performative traditions

*Qayapu* practitioners, both women and men, incorporate a Catholic religious foundation into their practice alongside symbolic relationships with the *Apus* (sacred mountains), shaping a culturally situated healing practice characterized by syncretism. During the ritual, specific actions are performed, such as holding the patient's head—generally that of a child—and reciting prayers addressed to different saints, depending on the specialist's beliefs (Figure 2). One female healer stated: “The child or the person does not get better even with the doctor; even doctors send them to me to perform Qayapu because their condition does not improve. People of all ages come because they have fallen or adults have had accidents; their bodies become disordered; they lose the desire to do anything. If there is no cure, they can die; they may become mute, foolish, or crazy.” This testimony illustrates how the practitioner understands and attributes therapeutic significance to Qayapu in addressing certain conditions, as well as its reported recognition within informal biomedical networks, without implying an assessment of its clinical efficacy. It reflects a culturally situated etiology in which events such as falls or accidents generate an imbalance between the body and the soul, affecting both children and adults.

**Figure 2:**
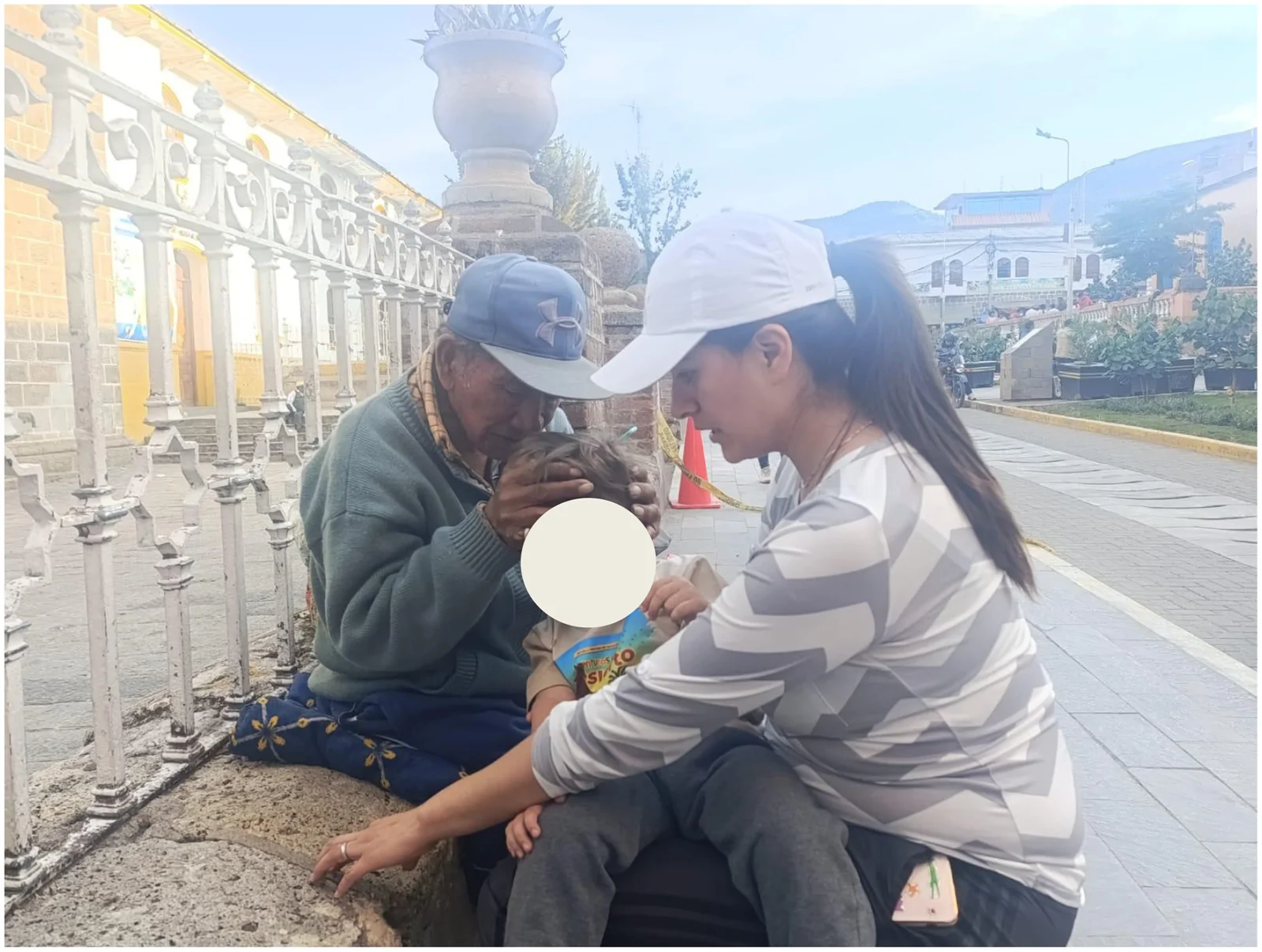
One of the healers who practices Qayapu provides services daily, from Monday to Sunday, in the atrium of San Francisco de Asís Church, located in the center of the city of Ayacucho. This is an area with heavy pedestrian traffic, where specialists set up their practice—generally using plastic chairs—and maintain a regular clientele. Photograph by the authors, January 2026.

The participation of women as therapeutic agents acquires particular relevance. As Velázquez and Ruiz Bravo (2024), point out, “in this case, Quechua culture allows for the recognition and appreciation of women's knowledge, experiences, and practices, in which art and rituals connected to nature play a leading role” (p. 172). This perspective makes it possible to understand that women's knowledge is not only culturally legitimized but also constitutes a central axis in the reproduction and transmission of ritual practices, where art and the relationship with nature contribute to the meanings attributed to healing processes.

Qayapu specialists, both in Andean communities and in urban settings—particularly in the city of Ayacucho—maintain that their practice does not operate according to a market logic but rather according to principles of voluntariness. Direct payment for the service is considered incompatible with ritual efficacy, since the deities would not accept the prayers, thereby affecting the healing process. In rural areas, compensation is usually expressed through non-monetary exchanges, mainly food, in accordance with reciprocity practices characteristic of Andean culture. The perceived outcome of the ritual is associated, according to the participants, with the confidence of the specialist and the faith of the person seeking help. One testimony expresses this as follows: “The child gets better, their appetite improves, they feel lighter, and their face changes. There are people who seek me out; there are people who believe because it truly heals what the hospital cannot.” This statement reflects the participant's perception of improvement and contributes to the construction of Qayapu's social legitimacy in relation to the limitations attributed to the hospital system.

Specialists identify specific bodily indicators—such as sunken or withdrawn eyes—as signs associated with *mancharisqa* (*susto*), distinguishing this culturally defined condition from ailments understood within the biomedical sphere. This distinction reflects the coexistence of different explanatory frameworks for illness, in which *mancharisqa* is understood through local concepts that are not necessarily aligned with biomedical interpretations. These practices are grounded in principles of the Andean worldview, in which “balance with Pachamama, prioritizing life, unity and integration, reciprocity, achieving consensus, among others, are highly valuable for the construction of *living well* within the community, understood as coexistence within a framework of respect, harmony, and balance” (Flores Gutiérrez et al., 2021, p. 99). Such principles guide community life and sustain the legitimacy of therapeutic practices based on balance, reciprocity, and collective harmony.

The head constitutes the central focus of the ritual, as it is locally conceived as the body's center of control, where the soul is understood to regulate bodily functioning in relation to balance. Although specific ritual spaces exist in the Andes, in the city of Ayacucho—the focus of this study—the practice is located in the urban center, particularly in the vicinity of a church, demonstrating a spatial reconfiguration of the ritual. The treatment is not limited to children; it also includes adults who place their trust in Qayapu (Figure 3). In many cases, these users are associated with migratory processes through which ancestral knowledge has been transferred, maintained, and reconfigured within urban environments. Practice requires patience and willingness on the part of the person seeking care, while specialists demonstrate knowledge of the ritual by identifying specific areas of intervention on the head. During the ritual, some participants were observed to enter states of deep relaxation or drowsiness.

**Figure 3:**
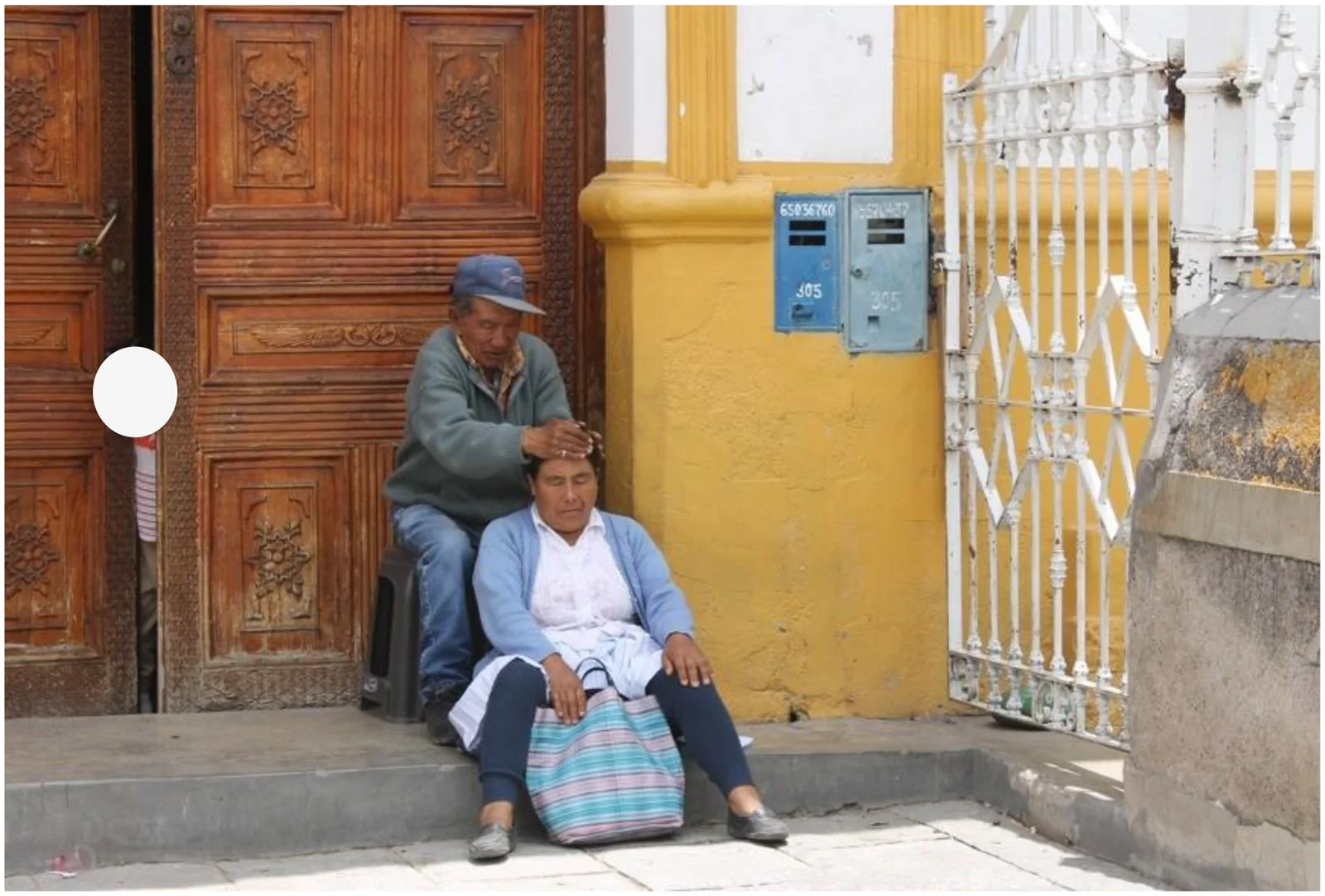
Adult woman undergoing healing through Qayapu, located at one of the side entrances of San Francisco de Asís Church. The participant chose a more private setting for the performance of the ritual, unlike the specialist's usual location. The specialist demonstrates flexibility in providing care, relocating according to the user's preference and even conducting interventions at patients' homes. Photograph by the researchers, January 2026.

In accordance with ethnographic tradition, the principal investigators participated in the healing ritual with the consent of the practitioners and while respecting the cultural protocols associated with the ritual. This participation facilitated the development of trust and enabled an experiential understanding of the meanings embedded in the ritual. It is acknowledged that the researcher's presence and involvement may have influenced both interactions with participants and the interpretation of the data. To reduce this potential bias, a reflexive field diary was maintained throughout the ethnographic research process, documenting personal reflections, methodological decisions, and possible analytical assumptions. Emerging interpretations were continuously contrasted with interviews, participant observations, and field notes, and were discussed with the co-authors to strengthen the credibility of the findings and the recognition of the commitment and seriousness with which practitioners carry out their healing practices.

One specialist described part of the procedure: “Sometimes they pass an egg over the person, then take it to the river where the water is flowing, and they have to collect the water against the current, and with that they perform the Qayapu. In the morning, when the sun is rising, it is effective. It may seem unbelievable, but you get healed.” This testimony highlights the use of natural elements, such as flowing water and a chicken egg, as well as the importance attributed to specific temporal conditions, such as dawn, within the ritual sequence. The collection of water against the current and its subsequent use in the ritual reinforce the symbolic dimension of these practices. These actions can be interpreted through a relational ontology, in which “the starting point is the conception of the origin of everything as a dichotomy or duality, where in the beginning there are two different elements, two essences that, among other characteristics, are complementary and proportional” (Drexler et al., 2015, p. 92). This perspective makes it possible to understand the ritual as structured around principles of complementarity, in which contrasting elements—such as current and countercurrent or heat and cold—are understood as participating in a dynamic balance.

Community narratives attribute significant therapeutic value to Qayapu in the healing of both children and adults. The term Qayapu refers to a culturally situated ritual practice aimed at calling back the soul, while the associated imbalance between soul and body is locally explained in relation to frightening experiences and other external factors. This recognition coexists with differing interpretations of Qayapu, particularly among evangelical groups, who question its cultural and ritual legitimacy. One interview illustrates this tension: “For example, I do not believe in it, but she does [referring to her neighbor]. From the day I committed myself to the Gospel, that evil spirit no longer affects me; thanks to God, even if I sleep in distant or deserted places, nothing happens to me. Before, when I was not evangelical, I was affected by it [*susto*]: I had to take a black dog, a black cat, and perform a *pagapu* [offering to the earth] with blood; otherwise, I would not recover.”

“Before knowing Christ, we believed in that; so, the devil also acts. She is still not evangelical and continues to believe.” The testimonies reveal a negotiation of religious legitimacy between evangelical and Catholic perspectives, with the latter showing greater affinity with practices such as Qayapu. In this sense, “the rapid and sustained growth of the Evangelical Church in Peru reflects its consolidation with its own rituals that downplay and eradicate, that is, extirpate, ancestral Andean practices” (Gutiérrez-Gómez et al., 2023, p. 2). This process can be interpreted as a dynamic negotiation of religious legitimacy in which evangelical perspectives challenge and, in some cases, contribute to the reconfiguration of ancestral Andean practices through alternative religious frameworks.

### 3.3 Therapeutic universality and intergenerational continuity of care

Adult participants and mothers place their trust in Qayapu as a culturally situated practice for addressing perceived imbalances between the mind and body, performed by specialists recognized within the community for their knowledge and experience. The ritual follows recurring patterns: the intervention focuses on the person's head and is accompanied by prayers and gentle rubbing, with the intention of calling the soul back to the body (Figure 4). The spoken discourse is: “*wawapa alman maipim kanky, kutimuy; kaikpi suyasunki cuerpuyki auuuuu kutirimuy*.” Translated: “Where are you, baby's soul, come back; your body is waiting here [prolonged shout: auuuuu], come back now.” Mothers of children who underwent the ritual described perceived changes following Qayapu, including improved sleep during the journey home. The manifestations associated with this condition include insomnia, persistent crying, diarrhea, sunken eyes, headache, and nocturnal fright responses, which practitioners and family members interpret as signs of *susto* or *mancharisqa* and address through Qayapu.

**Figure 4:**
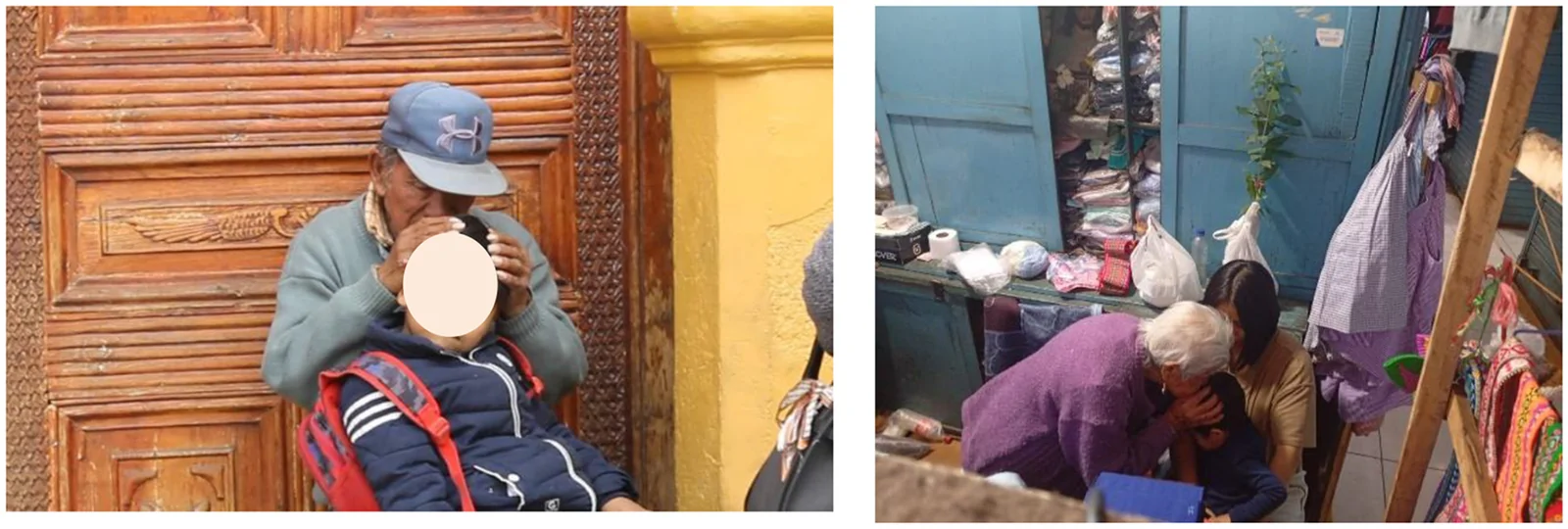
Schoolchildren undergoing Qayapu ritual at the entrance of San Francisco de Asís Church, located in the center of the city of Ayacucho, and at the central market. This space is recognized by users as a reference point for accessing specialists with gifts inherited from their ancestors. Photograph by the researchers, February 2026.

A specialist describes the scope of the ritual: “It is the healing of fright; only their little head is prayed over. To remove fright, it does not matter whether it is big or small, it is removed in all cases, even when it is strong. Their eyes become sunken, their little face looks wrinkled, they turn yellow like that; because they have fallen, because dogs bark and frighten them. To heal them, it is done two or three times, depending on how the person feels when they come. There are specific days and hours, at midday on Tuesdays and Fridays. It is better when it is done in silence, not in noise.” The ritual follows specific temporal patterns, including preferred days and times, as well as environmental conditions such as silence that are considered conducive to its performance. These features, rooted in an Andean cultural framework, continue to be practiced in urban spaces, including central areas of the city.

Scientific literature and mental health professionals have given limited attention to the ancestral knowledge associated with Qayapu, which is predominantly transmitted orally in Quechua and practiced within the same linguistic and cultural framework. In the Andean setting, experiences understood locally as forms of distress involving the relationship between mind and body are interpreted through a culturally specific system of knowledge that does not necessarily correspond to conventional psychological categories. In this sense, “Psychologists from around the world have been called upon to try to understand Indigenous peoples, when it would have been easier to consult Indigenous people themselves, their elders, their healers” (Pavón-Cuéllar, 2025, p. 103). This perspective highlights the importance of recognizing local knowledge as a legitimate framework for understanding experiences of illness and healing that are culturally situated.

The interpretation of these experiences through psychological frameworks may be limited, partly because Quechua predominates as the language of interaction and many knowledge holders, particularly older adults, have limited access to formal literacy and institutional forms of knowledge. One testimony illustrates the procedures and resources employed: “they have vomiting, diarrhea, the heart beats fast, they feel frightened, they do not eat. I chew coca, then a bundle of carnations, then egg, alum stone, only for Qayapu. I pray and recite the Our Father; Hail Mary; Holy Mary, I ask the Lord to heal their illness.” The ritual incorporates natural elements available in the local environment, including coca. As Cabieses (2018, p. 30) notes, “The consumption of coca leaves as a stimulant and anti-fatigue substance is found almost exclusively in Andean ecological zones above 2000 meters, where migration is intense.” Specialists consistently incorporate coca into Qayapu rituals when addressing conditions locally associated with *susto* or *mancharisqa*.

The community plays a central role in care processes, as families remain connected to their social environment through practices of visitation, mutual support, and solidarity, particularly in the transmission and practice of traditional forms of knowledge and healing. This social framework reinforces the perceived therapeutic value of Qayapu, which is evaluated by community members through their own experiences and observations. A specialist states: “They themselves call me, they tell me that they already have an appetite, that they are eating, that they no longer have fever; as soon as they wake up, they already want to eat. I tell them that for it to be effective it must be done for three consecutive days.” This statement illustrates how the specialist and family members assess the perceived outcome of Qayapu through observable changes in the child's physical and behavioral condition. Follow-up by family members, particularly mothers, constitutes an important component of the care process, as daily observation allows them to monitor changes in the child's condition.

This process is embedded in a cultural understanding of illness, where “the loss of the soul may result from inappropriate interactions with human or supernatural beings, such as encountering the wandering soul of a condemned sinner or fighting with relatives or neighbors” (Greenway, 1998, p. 10). Within this cultural framework, soul loss is understood in relation to social conflicts and encounters with supernatural beings, reflecting a relational, moral, and cosmological understanding of human imbalance. The return of the soul is understood by family members through visible changes in the child, which they interpret as signs of improvement. A mother states: “Their little face changes, you can see it changing; they already look healthy.” These expressions illustrate how family members interpret visible changes in the child as signs of restored balance, integrating physical, spiritual, and social dimensions within their understanding of wellbeing.

Qayapu is recognized by community members as a legitimate form of local knowledge, embedded within an Andean epistemological framework and a culturally specific understanding of illness and healing. Its foundation is linked to the conception of souls as entities with moral agency, insofar as “souls are protectors and, if not properly attended to, punishers. They are an ambivalent presence: ‘Souls protect us and also harm us”' (Aláez García, 2001). Within this cultural framework, souls are understood as ambivalent entities that may protect and sanction, with their significance embedded in a moral and relational understanding of social life. The social legitimacy of Qayapu is grounded in community recognition, which is sustained through shared experiences, continued practice, and intergenerational transmission of local knowledge. This recognition is expressed through collective attention to caregiving practices, whereby community members may question the absence of Qayapu in cases of childhood illness.

Regarding the ritual procedure, an interviewee states: “They take them to the place where they got frightened, and there they perform Qayapu; they gather it there, it seems like their spirit has stayed there. From there they bring it back to the body with Qayapu, and they follow the path where the person walked. Then they become calm again and start speaking.” This describes a ritual sequence that involves returning to the place where the fright occurred, where the soul of the child or adult is locally understood to have become detached from the body or to remain there. Within this cultural framework, the condition is understood as a separation between the soul and the body, and Qayapu is performed as a ritual process intended to call the soul back.

### 3.4 Inheritance, specialization, and the symbolic legitimacy of ritual knowledge

The Qayapu ritual, traditionally practiced in the Peruvian Andes, has undergone a process of relocation to urban spaces, particularly in the city of Ayacucho, where it is practiced in the historic center and in the atrium of a frequently visited church. Approximately ten older adults, predominantly men, were identified as specialists who practice Qayapu, understood locally as a form of soul-calling. Access to this practice involves a nominal monetary contribution ranging from 5 to 10 soles; however, practitioners emphasize its voluntary nature and do not establish fixed fees. Interaction preferably takes place in Quechua. The person seeking Qayapu asks, “*qaykam cuistan qayapu*,” and the specialist responds, “*voluntaumi mamay*,” translated as “How much does Qayapu cost?”—“It is voluntary, ma'am.” This form of exchange reflects a local understanding in which the perceived significance of the ritual does not depend on a formal economic transaction but on the voluntary contribution of the person seeking Qayapu, since specialists describe their spiritual gift as non-negotiable.

In the urban setting and its surroundings, older women were also observed (Figure 5) practicing Qayapu on a voluntary basis, within a spiritual framework in which a divine entity is invoked as part of the ritual intended to call the child's soul back. These specialists, who are socially recognized for their experience, play a central role in the continuity of this ritual practice. Access to these specialists is facilitated by relationships of trust and social recognition. There is reluctance to perform Qayapu for unknown individuals, as expressed in the statement: “*pim nisunky ñuqa qapaq kani nispa, manam yachanichu, sasam chaiqa burlachuqaya*,” translated as: “Who is the person who told you that I know how to do Qayapu? I don't know anything; they say it just for fun. This is not a joke; it is done properly.” This response illustrates mechanisms for safeguarding ritual knowledge and regulating access to specialists, whereby referral through a known third party helps establish trust and the seriousness attributed to the request for Qayapu.

**Figure 5:**
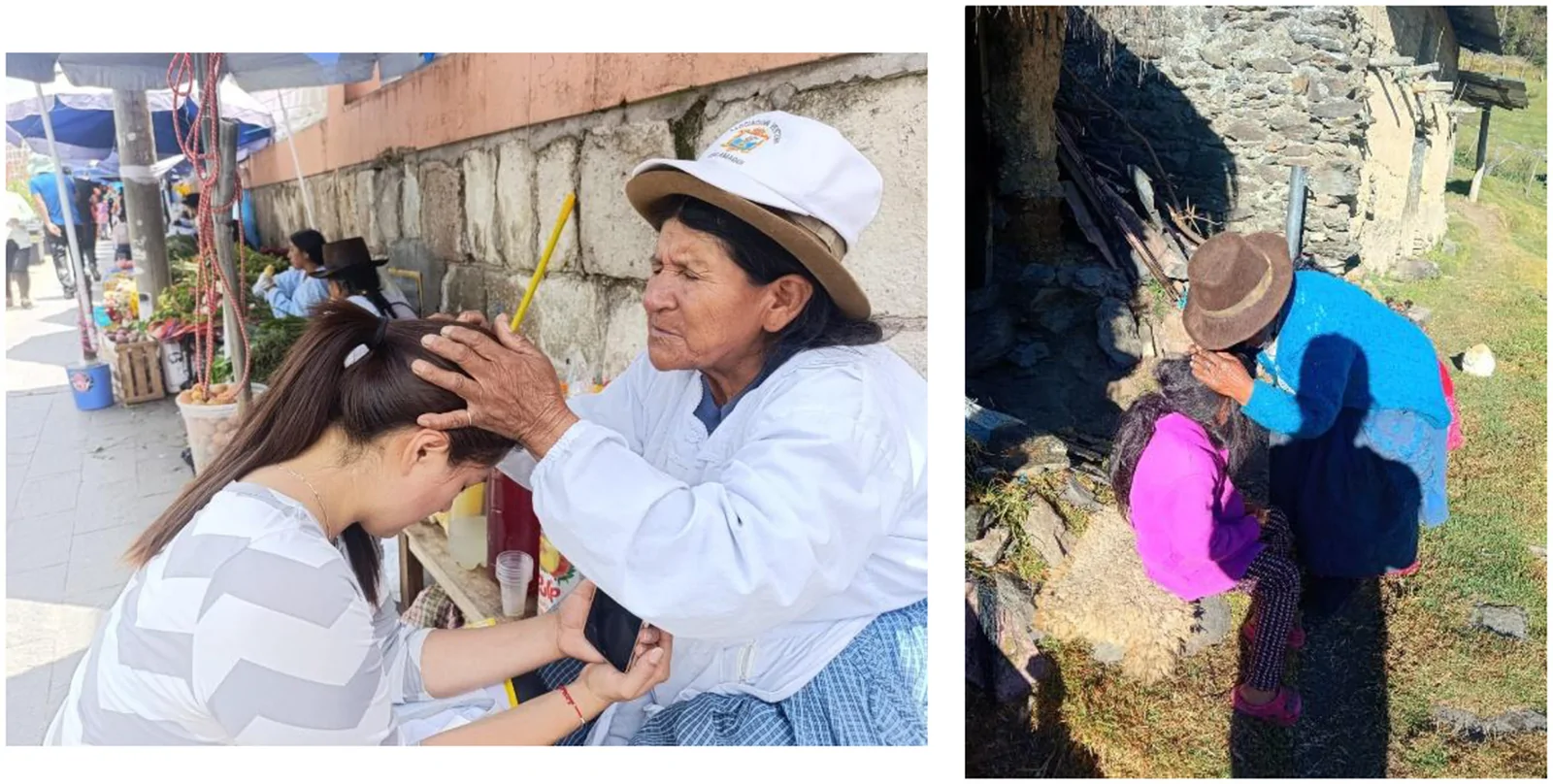
Record taken in the surroundings of the Central Market in the city of Ayacucho. Older women were observed performing Qayapu on a voluntary basis, receiving people mainly through referrals from acquaintances. One of the researchers was able to participate in the ritual as a person seeking Qayapu, with the specialist's authorization. Photograph by the authors, January 2026.

The interactions observed in the urban setting did not indicate a clearly profit-driven logic in the specialists' practice. Economic compensation is voluntary and non-standardized: practitioners accept the amount that the person seeking Qayapu—or, in the case of minors, the mother—decides to give. No practices of negotiation or explicit fee-setting were recorded, and the practice is carried out discreetly. This finding can be interpreted in light of what Michael M. J. Fischer (2023, p. 3) argues, when he states that “as a response to globalization, ritualism became a vehicle both for the commodification of vernacular culture and for political ethnization”. Ritualism can operate as a mode of adaptation through which local practices are incorporated into broader social dynamics, potentially acquiring new symbolic meanings while also serving as resources for identity affirmation.

In the case analyzed, this potential commodification is not explicitly observed but coexists with an ethic of gift and vocation. Specialists emphasize the continuity of the practice through transmission and cultural legitimacy rather than economic gain. A female specialist states: “the way of doing it has not changed, but the number of people seeking Qayapu has decreased; they no longer believe, most of them take their children to the hospital. Not just anyone can do it; one must learn and know the prayers; it is like a special gift.” A recurrent concern among specialists is the gradual loss of cultural heritage as biomedicine becomes increasingly central to healthcare. Despite this, the ritual practice persists in the urban environment as an expression of intergenerational continuity and cultural resilience, maintaining ancestral forms of healing that have historically contributed to community wellbeing.

No heirs of a strict shamanic tradition are identified, nor do the actors self-designate as healers. They identify themselves as specialists who have received a “gift” transmitted mainly through family lineage, from parents to children or other close relatives. The transition toward specialization does not follow formal initiation processes but rather develops through repeated requests from people seeking Qayapu, who contribute to the social recognition of the knowledge associated with the family lineage. It is relevant to decouple the analysis from a classical conception of shamanism, considering that “both the practicing shaman and their audience; and, finally, the fact that indigenous shamanism ‘works' in transcultural contexts, as it increasingly serves non-indigenous patients, which implies additional processes of cultural translation” (Taylor, 2014, p. 97). Shamanism can thus be understood as an adaptable system that mediates meanings between specialists and their audiences and may extend beyond indigenous communities through processes of cultural translation.

The legitimacy of specialists is constructed through circuits of oral transmission and community recognition. Knowledge and reputation circulate through interpersonal networks without institutional mediation. A female specialist states: “I learned on my own by watching, 7 years ago. People already know me and send their children or come on their own because I heal; everyone calls me Mrs. Gerarda.” This testimony illustrates a process of self-learning based on observation and the meaningful appropriation of prayers, which are considered a central component of Qayapu.

Knowledge of the ritual processes involved in soul-calling is understood as essential to the proper performance of Qayapu. The omission or improper execution of these procedures is associated, according to informants, with the failure to restore the patient's balance or with incomplete recovery. According to informants, failure to follow the established ritual sequence may affect the specialist's legitimacy and, consequently, their social recognition and the continuity of their practice. The continuity of cultural heritage is sustained by the perceived effectiveness of the treatment, particularly in the resolution of emotional imbalances that affect the relationship between body and soul.

This finding is consistent with what is indicated in the literature: “there appears to be a theoretical consensus on the vital importance of space and rituality for the configuration of Andean societies, as well as the historical variability and perspectives of studies” (Muñoz-Henríquez et al., 2023, p. 2). This consensus highlights the centrality of space and rituality, even though interpretive diversity persists across academic approaches. Relative homogeneity is also identified in therapeutic forms of intervention, characterized by the centrality of prayer and bodily contact. As one practitioner states: “I do it only with prayer; I make them sit down and I pray while holding their head.” Methodologically, the data were collected in Quechua; however, responses tend to be brief and evasive, which introduces limitations to interpretive depth and to the expansion of the ana.

### 3.5 Reimagining Andean healing in conjunction with the biomedical system

Duality is identified in the treatment of emotional imbalance between body and soul in children. Interviews with adult patients and mothers report therapeutic efficacy in the procedures applied. Distrust of conventional pediatric care is recorded, attributed to the prescription of drugs (pills or syrups) that children tend to reject, which constitutes an initial factor for seeking Qayapu specialists. A therapeutic modality applicable to both children and adults is documented, based on the use of plant elements and prayer. A specialist states: “in children it is clavel [carnation flower], there are some herbs that are called *mancharisqa* [frightened]; for example, with a bunch of clavel they pass it over the whole body, praying the Our Father, then they finish passing it and the clavel is broken and tied to the head when the child is already in bed; the next day when the child wakes up, it is removed and if there is a garden, flowerpot, or field, it is thrown there.” The testimony indicates that the plant elements are incorporated into a ritual sequence that combines bodily contact, prayer, temporary placement of the carnation on the child's head, and its subsequent removal and disposal. Rather than being presented by the specialist as isolated therapeutic substances, these elements form part of a practice directed toward restoring the body–soul balance associated with *mancharisqa*. A predominance of prayers associated with the Catholic religion is observed.

The intervention on the head, carried out with patience and devotion, is configured as the operational core of the treatment, with effects interpreted as relaxation. This procedure is linked to the notion of *susto*, defined as “a type of intense trauma that some people experience when they are startled by the unexpected presence of animals, people, shadows, or spirits” (Calderon-Torres et al., 2023, p. 173). In the present study, however, *susto* is understood not as a single or universal form of fear, but as a culturally situated experience in which fright is associated with an imbalance between body and soul and is interpreted locally through specific physical, emotional, and behavioral manifestations. The body–soul imbalance resulting from *susto* is addressed through specialized Qayapu.

Social interaction enables the identification of observable indicators in children, such as alterations in facial expression (particularly in the eyes and face), the presence of diarrhea, loss of appetite, and episodes of persistent crying without an apparent cause. In the face of these manifestations, the surrounding community interprets the condition as *mancharisqa*. Multiple testimonies report perceived therapeutic efficacy, associated with the child's recovery following the Qayapu procedure. In symptomatic terms, a specialist states: “they cry a lot, they cannot sleep because of fright, they have no appetite; all of that goes away until they are cured, until Qayapa is performed, and then the children return to normal.”

A recurrent questioning of hospitalization processes is documented. Even within hospital settings, some users suggest as an alternative going to the entrance of the Church of San Francisco de Asís and the surroundings of the Central Market, the location of the study. This practice is consistent with what is indicated in the literature: “from a community perspective, it can be observed how the Andean medical system maintains its vitality and relevance today. An illustrative case of this continuity is the district of Quinua, located approximately 50 min from the city of Ayacucho” (Ramos Lopez et al., 2025, p. 2). In line with these findings, the present study shows the continued presence of the Andean therapeutic system in an urban context, specifically in the city center of the Ayacucho region.

The treatment evidences an epistemological conflict in the therapeutic decision-making of mothers, the main users of Qayapu specialists. Faced with the urgency of resolving acute conditions in their children, they prioritize outpatient care based on the “calling of the souls.” This preference coexists with distrust toward biomedical medicine, while conversely, from the professional medical field, the therapeutic validity of Qayapu is questioned, particularly in its psychological dimension. During data collection, the testimonies consistently associated *susto* with the loss or displacement of the spirit and with a set of physical and behavioral manifestations. A specialist states: “it is so that their spirit returns; prayers are said, the calling is performed. When children fall, they get frightened, they do not sleep, they do not eat, they have no appetite, they only want liquids, their stomach becomes loose, sometimes they have vomiting, fever, and above all they do not sleep; they do not even want food, only water.” This symptomatic repertoire articulates physical and behavioral signs that, from a biomedical perspective, may warrant pediatric evaluation, while within the local therapeutic framework they are interpreted in relation to *susto* and addressed through ritual intervention, including prayers and manual manipulation of the head.

The coexistence of hybrid therapeutic itineraries, in which illness is initially interpreted as a common ailment and treated with domestic or pharmacological resources, and later—or simultaneously—incorporates the spiritual dimension: “the illness is first treated as a common ailment, not as a spiritual illness, and herbal or pharmaceutical remedies are administered sequentially or simultaneously depending on the patient's symptoms” (Calvet-Mir et al., 2008, p. 4). The conflict between traditional medicine and biomedicine should not be understood as a dichotomous opposition, but rather as a structural tension between different systems of knowledge, interpretation, and healthcare provision.

While Indigenous healing practices hold significant cultural legitimacy and continue to play an important role in Andean communities, their therapeutic practices are grounded in explanatory frameworks that differ from those of biomedicine. In this sense, the coexistence of Qayapu and biomedical care reflects different ways of interpreting illness, distress, recovery, and the relationship between body and soul. Biomedicine may address physical and clinical dimensions of illness, whereas Qayapu incorporates cultural, emotional, and spiritual dimensions that patients and practitioners consider relevant to the healing process. The articulation between these systems therefore requires an anthropological approach that recognizes their distinct therapeutic rationalities and the ways in which people navigate between them.

Despite the availability of biomedical services and health posts throughout Peru, the belief persists that the soul can become detached from the body following an adverse event, generating an emotional imbalance whose treatment is attributed to the Qayapu ritual performed by a specialist. It is necessary to acknowledge the concerns of parents and patients regarding the need to navigate both healing approaches without discrimination. The analysis of *susto* and its origins reveals a set of clinical manifestations described as “loss of motivation, apathy, crying, restlessness, introversion, deterioration, sadness, loss of appetite, sleep problems, and, in severe cases, delusions and hallucinations, which could link susto to major depressive disorder” (Martínez-Radl et al., 2023, p. 697). However, *susto* should not be treated as a homogeneous or universally defined condition. Anthropological research has questioned the assumption that experiences described as *susto* necessarily correspond to a single form of fear or a standardized clinical entity, emphasizing the importance of examining how fear, distress, and illness are locally conceptualized (Gamlin et al., 2020; Waldram and Hatala, 2023). In the present study, *susto* is understood as a culturally situated experience of fright associated by Qayapu specialists and users with the loss or displacement of the spirit and with physical, emotional, and behavioral manifestations. This conceptualization does not imply equivalence between *susto* and a psychiatric diagnosis; rather, it highlights the need to examine the local meanings through which distress is recognized, interpreted, and treated in the Andean therapeutic setting.

## 4 Limitations

This study presents several limitations. The purposive sample was relatively small and was restricted to the urban context of Huamanga; therefore, the findings are not intended to be statistically representative or generalizable to other Andean contexts. In addition, *Qayapu* therapeutic practices vary according to geographical and sociocultural contexts; consequently, the modalities observed in urban settings may differ from those developed in rural communities, where the ritual incorporates other actors, spaces, and symbolic elements. The ethnographic approach and participant observation imply that the researcher's presence may have influenced certain interactions and narratives; this potential bias was mitigated through prolonged field engagement, data triangulation, and discussion of the analytical categories with the co-authors. The interviews conducted in Ayacucho Quechua were carefully translated into Spanish in an effort to preserve their cultural meanings; however, some linguistic and symbolic nuances may not have been fully conveyed.

## 5 Conclusions

In the process of modernization and universalization of mental health services, the belief in Qayapu (soul calling) persists, attributed to episodes of *susto* or intense fear, generally of a fortuitous nature. This condition is interpreted as an emotional imbalance that compromises the relationship between body and soul, affecting both children and adults. The treatment is based on a symbolic therapeutic device performed by a healer with inherited gifts—mainly through family lineage—whose legitimacy rests on the intergenerational transmission of knowledge. The healing ritual involves the laying on of hands over the patient's head and the recitation of prayers directed to the ñ*utqun* (brain), invoking Catholic saints, which evidences a syncretic process characteristic of Andean culture. Due to internal migration dynamics, this practice has shifted to urban spaces in the city of Ayacucho, taking place in the historic center and in public areas of pedestrian and vehicular traffic, particularly in the atrium of the colonial Church of San Francisco de Asís.

A tension is identified in the perception of therapeutic efficacy between practitioners and believers in soul-calling (*llamado de las almas*) and health professionals who question its validity. Observable signs in children—facial pallor, sunken eyes, unexplained crying, diarrhea, loss of appetite, and sleep disturbances—are locally interpreted as *mancharisqa* (state of fright) or *llumpay mancharisqa* (deep fright), which leads to the indication of Qayapu as treatment. Immediate social networks (neighbors, relatives, and acquaintances) intervene in therapeutic decision-making, encouraging consultation with a healer—a preferred designation over “curer” or “shaman”—generally an elderly person, without distinction of sex. Service provision is framed within a logic of voluntariness: no prior fee is established, and compensation is left to the later discretion of the patient or their relatives. Male participation in this practice predominates. The absence of fixed prices is justified by the premise that the effectiveness of the treatment would be compromised if the gift—conceived as bestowed by God—were subject to economic negotiation.

Female healers carry out their practice in a complementary manner alongside small-scale commercial activities, mainly in spaces such as the market. Care is mediated through trusted intermediaries, which delimits access and reinforces the perceived legitimacy of the treatment. The presence of unknown individuals is avoided; when direct requests for care are made, the response is typically closed—*manam yachanichu* (I do not know, nothing)—with no openness to dialogue or negotiation. When interpersonal trust exists and belief in the efficacy of *susto* treatment is present, a critical view of biomedical practice emerges, particularly regarding the prescription of medications (pills and syrups), which are considered insufficient or even harmful in children's recovery. Despite processes of social change and the expansion of health services, the practice of soul-calling remains active as a therapeutic resource. Its presence in urban spaces demonstrates the continuity and adaptation of this tradition within modern contexts.

There is a need for systematic research on the psychological efficacy of prayer applied to the patient's head—an area associated with the brain—during the Qayapu ritual, considering that it is frequently reported that children fall asleep during the intervention. The incorporation of this knowledge into the educational curriculum is proposed from an anthropological perspective, along with the analysis of hybrid care models that articulate conventional medicine and ancestral knowledge, with the aim of contributing to the reduction of child mortality and preventing possible adverse effects derived from exclusively symbolic interpretations of Qayapu. It is also necessary to further examine the meaning of prayers and the therapeutic practices carried out by older adults, given that no significant participation of younger generations is observed in the continuity of this cultural heritage.

## Data Availability

The original contributions presented in the study are included in the article/Supplementary material, further inquiries can be directed to the corresponding authors.
