# Supplementary material for: Qayapu in the reconfiguration of Andean healing and the ritual recovery of the child's soul
Source: Front Sociol. 2026 Oct 20;11:1870310. doi: 10.3389/fsoc.2026.1870310 (PMC13727419; doi:10.3389/fsoc.2026.1870310)
Supplement: Supplementary file 1 [file Data_Sheet_1.pdf]

Supplementary Material

1 Supplementary Data

SUPPLEMENTARY INTERVIEW DATA

INTERVIEW 1

Mr. Víctor, 75 years old, originally from Huamanga, currently lives in Huascahura. He attends at "the arch" from Monday to Saturday, 9:00 a.m. to 7:00 p.m., although he often leaves earlier. He has been working in the same place for 28 years. People now seek him out because he is the only one left; before, there were three elderly practitioners, but during the pandemic they disappeared—perhaps they died or were taken back to their hometowns. Mr. Víctor treats not only babies and children, but also adults and the elderly.

What is the qayapu?
"It is fright (susto) that makes a person's little soul ill. For that, we pray so the person can heal. The soul does not leave or come out of the body—if it did, we would die. We only call it so it can be healed. If we do not pray and it is not cured, the person can even go mad."

From what age is the qayapu performed?
"The qayapu is done from newborns up to 80 years old—everyone comes: mothers, adults, children, even the disabled."

Who should be present during the qayapu?
"For children, their parents must be present. Adults can come alone. The important thing is that the patient comes."

In what situations does fright occur and require the qayapu?
"Yes, when they fall, when they get scared—by dogs, cats. Children are afraid of animals. People also bring their children when something has happened in the hills and they want me to go there or do it in silence, but I don't do that. I attend only here; there is no difference in the effect, and I would lose time going there."

What symptoms do they have?
"They have vomiting, diarrhea, their heart trembles, they feel timid and frightened, and they don't eat."

What do you use to perform the qayapu?
"I use coca leaves, I chew coca, then a bundle of carnations, then an egg, and an alum stone—nothing more. I pray the Our Father, Hail Mary, Holy Mary—only to the Lord so that He heals them."

How do you know the soul has been healed?
"I have to treat them up to three times because it is the devil who causes the fright. After three times it passes. The first time they calm down; the second and third time they feel better."

How did you learn to perform the qayapu?
"I learned from my father and my mother. I memorized what they did. My father, Juan de Dios Izaguirre Ayme, and my mother, Celestina Sauñe Porres, are now deceased. They always practiced it, and people sought them out."

Has the way of performing the qayapu changed?
"It has not changed. What has decreased is the number of people who seek it—they no longer believe as much. More people are taken to the hospital now."

Do you think anyone can perform the qayapu?
"Not just anyone can do it. You must learn and know the prayers; it is like a gift."

Analytical note:
This testimony shows how knowledge of the qayapu is transmitted within the family sphere and how, despite social and cultural changes, it continues to be practiced as a form of traditional healing within the community.

INTERVIEW 2

Mrs. Marina, 89 years old, originally from Huamanga, has a stall inside the Central Market where she sells baby clothes and sweaters knitted by herself. She is well known for performing the qayapu and has been practicing it for more than 40 years.

What is the qayapu?
"It is done so that the spirit returns. We pray and call it back. When children fall or get frightened, they don't sleep, they don't eat, they lose their appetite, they only want liquids, their stomach loosens, sometimes they vomit, they get a fever, and above all they don't sleep. They don't want food, only water."

Why is it important to perform the qayapu?
"Because the child or person does not heal even with a doctor. Even doctors send them to me to have the qayapu done, because they don't get better. People of all ages come—those who have fallen or had accidents. Their body becomes disturbed, they have no desire for anything. If there is no cure, they can die; they may become mute, dull, or even mad."

In what situations do they get frightened?
"Babies fall from the bed, or some animal frightens them. They can be frightened by anything. For example, yesterday a boy came—his mother had watched a horror movie; he was already 16 years old. He was acting strangely, walking oddly, trying to jump. His grandmother brought him. I asked him to be honest with me: 'What did you see to be like this?' He said, 'My mother watched a horror movie, and now I'm like this.' And he wasn't even that young—see how it affects them."

How do you perform the qayapu?
"I pray the words of God."

The Our Father?
"No, no. I have other prayers. It is always directed to God, to the Virgin Mary, to all the saints—Saint John of God, Our Lady of Remedies, Martin de Porres. For me, they are doctors. There are others who only say 'cutimuy' (return to your body)."

What do you use?
"Well, I do not use eggs or alum stone with children because it dehydrates them—that is for adults. With children I use carnations. There are herbs called *manchariscca* or *huahillall*. For example, with a small bunch of carnations I pass it over the entire body while praying the Our Father. Then the carnation is taken apart and tied on the child's head when they are in bed. The next day, when the child wakes up, it is removed and, if there is a garden, a flowerpot, or a field, it is discarded there. If there is nowhere to take it, the carnation is wrapped in newspaper and placed under the pillow; when it dries, it is thrown away. They are also given the herb *huahullall* to drink—those little remedies are taken."

Who should be present?
"Only me and the patient. When parents come, I ask them to wait aside."

How do you know when people have been healed?
"They call me themselves and tell me they have regained their appetite, that they are eating, they no longer have fever. As soon as they get up, they want to eat. I tell them that for it to be effective it must be done for three consecutive days."

From whom did you learn to perform the qayapu?
"From my grandmother Josefa. I liked it and learned, thanks to God, all her prayers."

Do you think anyone can perform the qayapu?
"Yes, anyone who truly knows and has learned. But there are people who just pray without meaning, or say words that make no sense. Once I was frightened by a car that almost ran me over, and I felt unwell. I prayed for myself. Here, on the steps of the market, they told me about an old woman who sold herbs. I went, and I don't know what nonsense she said—just a few words and that was all. I told her my hand had been hurt; she said she would pray not only for the head and charged 10 soles. I told her I would only give her 5 soles because I also perform qayapu and charge 6 soles. You are going to charge 10 soles for just a few words—that is not correct. That is why not everyone does it with the same faith."

Is the way of performing the qayapu the same?
"No, each person has their own way; it is different."

Do you think the number of people seeking the qayapu has decreased?
"No, it has not decreased. People always come looking for me—especially on Tuesdays and Fridays at exactly 12 noon, but they may come at any time."

Analytical note:
Mrs. Marina appears as a key transmitter of this tradition, showing how learning persists across generations (from her grandmother). Her practice is grounded in vocation rather than profit, and she differs from other practitioners by not using eggs with children, highlighting internal variation within the qayapu tradition.

INTERVIEW 3

Mrs. Gerarda, 56 years old, originally from Huamanguilla, sells vegetables on the street. People know that she performs the qayapu and seek her out for healing. She has been practicing the qayapu for 7 years.

What is the qayapu?
"It is the healing of fright (susto). We only pray over the head. It removes fright, whether it is small or large—it works for everyone. When it is severe, they also come."

When do they become ill?
"They have sunken eyes, their little face looks wrinkled, they turn yellow—like that they become."

Why do they get frightened?
"Because they have fallen, or because dogs bark and scare them. To heal them, it is done two or three times, depending on how the person feels. There are specific days and times—at noon on Tuesdays and Fridays. It is better when it is done in silence, not in noise."

What do you use?
"Only prayers and calling. Sometimes I pass an egg over them when it is severe, also herbs—*qapia qapia*—for them to drink, and that removes it."

How do you know they have been healed?
"Their face changes visibly—it looks healthy again."

From whom did you learn?
"I learned on my own, by observing, seven years ago. People already know me and send their children to me, or they come by themselves because I heal. Everyone says, 'go to Mrs. Gerarda.'"

Do you think people believe more in the qayapu or less now?
"Yes, more people come to seek me out, both adults and children."

Observation note:
The interview conditions were challenging due to street noise, as she sells vegetables and customers frequently approached to ask prices for corn and potatoes. This made the interview uncomfortable for her. Additionally, she primarily speaks Quechua.

INTERVIEW 4

Mrs., 83 years old, originally from Socos (Joya Joya annex), is located on the steps of the Central Market near the Arco de 28 de Julio. She is known for performing the qayapu and sells herbal preparations marketed as remedies for the liver, kidneys, and other ailments. *(The interview was conducted in Quechua.)*

What is the qayapu?
"Qayapu is a Quechua word that means the calling of the spirit. When a person is frightened, the Lord Jesus Christ returns our soul or spirit."

Why is it important to perform the qayapu for someone who is frightened?
"Yes, it is important because they truly need the qayapu. If they are not healed, they can even die. Children or people in that condition—many have been brought to me after being hospitalized for up to two weeks, and they have improved."

What happens to the person's soul when it is lost or frightened?
"The soul moves away; that is why it is necessary to take them to someone who performs the qayapu."

In what places or moments do these fright events most often occur?
"They can be frightened anywhere, but mostly in the fields—due to falls, animals, or at night. Those who are frightened in water become mute; they cannot speak."

How do you recognize that a person needs the qayapu?
"Because the child tries to sleep but cannot; they startle repeatedly and wake up crying. They develop fever, vomiting, do not eat, have diarrhea, and become very ill."

How do you perform the qayapu and what do you use?
"I do not use anything; I only use prayer because I am Evangelical. Catholics use candles, flowers, cigarettes, and other things. I mention the child's name and begin the prayer, always from top to bottom."

Where is it recommended to perform the qayapu?
"Anywhere—it can be done at home or in silence. I do it here in the street because I sell my products; people seek me out because they already know me."

Who should be present during the qayapu?
"For children, only their parents; for adults, they can come alone."

From whom did you learn to perform the qayapu?
"From my grandparents; they also performed the qayapu when I was a child."

Do you think anyone can perform the qayapu?
"Not just anyone can do it. One must have a gift, because we use prayer—the prayer of God—and it must be someone who has that gift with fervor, believing that healing will occur."

How do you know the soul has returned?
"The child begins to improve—they eat, feel lighter, and sleep better."

Nowadays, do people seek the qayapu more than before?
"Yes, in a day I may attend around 40 people or children. Each day is different; sometimes there are more, sometimes fewer."

Has the way of performing the qayapu changed over time?
"No, because I have always performed it through prayer; prayer has power."

Analytical note:
This testimony highlights the continuity of traditional knowledge and its ongoing relevance within the community, as well as the central role attributed to the spiritual dimension in healing processes. It also reveals internal tensions among practitioners: according to Mrs. Marina Rojas, this practitioner's method—based solely on brief prayer—would not constitute a "proper" qayapu, indicating variation and contestation within the practice.

INTERVIEW 5

Mrs. Margarita, 54 years old, originally from a village near Ayacucho, has lived in the city of Ayacucho for many years. She sells lemons and other vegetables near the Central Market, alongside her daughter, who has a stall next to hers. She has been practicing the qayapu for more than four years. *(The interview was conducted in Quechua.)*

What is the qayapu?
"It is the calling of the soul when a person is frightened."

Why is it important to perform the qayapu when someone is frightened?
"It is important because all children get frightened, almost always when they fall."

What happens to the soul when it is frightened?
"They usually develop fever, have headaches, become pale, have vomiting, and cannot sleep."

In what places do they most often become frightened?
"Fright is almost always caused by falls."

How do you perform the qayapu?
"I am a 'sister' (Evangelical believer). I only use prayer. Catholics use other things; I am Evangelical—I only pray and say the name of the child or the adult."

In what places is it recommended to perform the qayapu?
"Anywhere. There are no rules for performing the qayapu. I do it here in the street because people look for me and already know me."

Who should be present during the qayapu?
"Children come with their parents; if they are adults, they come alone."

From whom did you learn this practice?
"I was taught only by the Lord God."

Do you think anyone can perform the qayapu?
"Not just anyone. Since we use prayer, it must be someone who respects the word of God and has the gift to do it with faith."

How do you know the soul has returned?
"The child recovers—appetite improves, they feel lighter, and their face changes."

Nowadays, do people seek you more than before?
"Yes, there are people who come to me. Some believe because it truly heals what hospitals cannot."

Observation note:
This interview was particularly difficult. The participant spoke very little, providing brief responses, and felt uncomfortable with the camera. The main difficulty arose from her daughter, who objected to the interview, demanded payment for the information in an aggressive manner, and even shouted before the recording. This resulted in one of the shortest interviews. Additionally, the participant primarily speaks Quechua.

INTERVIEW 6

Mrs. Lucía, 78 years old, speaks very little Spanish. She is occasionally found next to her daughters' stall, where they sell potatoes. She mainly performs this practice within her family and reports that she has been practicing the qayapu since around age 40. *(The interview was conducted in Quechua.)*

What is the qayapu?
"It is the healing of fright. When children are frightened, we heal them."

Why is it important to perform the qayapu?
"It is important because all children get frightened, mostly when they fall."

What happens to people when they become ill or frightened?
"They usually have fever, vomiting, and headaches."

In what places or moments do they become frightened?
"Almost always from falls—children while playing, with animals, or in accidents."

How do you perform the qayapu?
"I only use water. I put water in a glass and sprinkle it over the head up to three times, while I say a prayer in their name."

How do you know the qayapu has worked?
"The child begins to improve—their face, which was pale, returns to normal; they start eating and sleeping better."

In what places is it recommended to perform the qayapu?
"In any place. I do it at home or even in the street."

Who should be present during the qayapu?
"Children should always be with their parents; adults can come alone."

From whom did you learn to perform the qayapu?
"I learned on my own, out of necessity for my child. I took him, saying he had been frightened and needed healing. She told me how to do it, and the next day my child improved. Since then, I have been doing it."

Do you think anyone can perform the qayapu?
"Not just anyone can do it—they must know how."

Nowadays, has the number of people seeking the qayapu increased?
"Yes, they always come looking for me."

INTERVIEW 7

Mrs. María, 83 years old, originally from Vista Alegre in Huamanga, has a stall inside a small market next to the Central Market where she sells ground products. She did not agree to be video recorded—only audio. She reports that she has been performing the qayapu for 20 years. *(The interview was conducted in Quechua.)*

What is the qayapu?
"It is the healing of the fright of the soul."

Why is it important to perform the qayapu?
"It is important because children get frightened."

What happens to the soul when they are frightened?
"They have fever, headaches, and vomiting."

In what places do they become frightened?
"Mostly from falls, while playing, and also in water."

What symptoms do they have?
"They have headaches, come with fever, and cannot sleep."

How is the qayapu performed?
"I do it only with prayer. I have them sit and say a prayer while holding their head."

In what places is the qayapu performed?
“In any place—they come to my home or here in the market.”

What do you use?
“Only prayer and the person’s name.”

Who should be present during the qayapu?
“Children with their parents; adults come alone.”

From whom did you learn to perform the qayapu?
“I learned on my own, using the prayers of my church. I am Evangelical, a believer in the word of God.”

Do you think anyone can perform the qayapu?
“Not just anyone can do it—they must know how.”

How do you know the soul has been healed?
“The child begins to eat, sleeps better, and improves.”

Do people still seek the qayapu?
“Yes, they still come to me.”

Observation note:
Due to her age, Mrs. María was quite reserved during the interview. It was not possible to explore further, as she declined video recording and limited her responses. The interaction was conducted entirely in Quechua, with very brief answers.

INTERVIEW WITH QAYAPU RECIPIENTS (“PATIENTS”)

INTERVIEW 1

Mr. Próspero, 63 years old, together with his wife Teodora Amiquero Durante, 58 years old, went to Mr. Víctor Izaguirre, who is located at the Arco de 28 de Julio. They kindly agreed to speak with us and shared their perspective based on their experiences with the qayapu.

What is the qayapu for you?
“It is when a person gets frightened—you become like mute, kind of slow, and every time you sleep you suddenly jump like this (he gestures a jumping movement). That is fright (susto); it means your spirit has moved away and is no longer there. Sometimes your eyes look deeply sunken. For example, my wife came because she gets frightened by everything—small noises make her jump. She was frightened after overturning on a motorcycle.”

Do you always practice this?
"Yes. Sometimes they pass an egg over the body; sometimes they take you to the river, where the water is flowing, and they must draw it back against the current, and with that they perform the qayapu. In the morning, when the sun is rising, it is effective. It may seem unbelievable, but you heal."

When your children get frightened, do you always bring them?
"Yes, we always accompany them. There is an even more effective qayapu when the fright is severe—when they lose speech completely. There is no cure, not even in the hospital. Do you know what must be done? You must take a practitioner like him (points to Mr. Víctor) to the place where the fright occurred. There, they collect it—meaning the spirit seems to have remained there. From there, they bring it back to the body through the qayapu, retracing the path the person walked. Then the person becomes calm, returns, and begins to speak again."

How many times must it be done to improve?
"When it is severe, three times; when it is mild, once or twice is enough."

Before coming for the qayapu, did you go to the hospital or come directly?
"No, we came directly, because the people at the health center do not know."

Do you think people should continue practicing the qayapu?
"That depends on each person. For example, I do not believe, but she does. Since the day I became Evangelical, that evil spirit no longer affects me—thanks to God. Even if I sleep in remote or deserted places, nothing happens to me. But before becoming Evangelical, it did affect me—I had to carry a black dog or black cat, and always perform *pagapu* with blood; otherwise, I would not heal. Before knowing Christ, we believed in that, so the devil also acts. She is not Evangelical yet and still believes."

So it depends on beliefs?
"Yes, it also depends on that, because it is the work of the evil one. When the spirit of God enters, everything changes."

Do you think young people still believe in the qayapu?
"Nowadays, young people do not believe anymore, because in the past our parents taught us how to heal."

Commentary note:
Mr. Próspero was very cooperative and provided extensive information based on his experience. His account reveals a clear contradiction: although he claims not to believe in the practice due to his Evangelical faith, he continues to take his wife and children to undergo the qayapu. This suggests a pragmatic recognition of its perceived effectiveness, even in tension with his declared beliefs.

INTERVIEW WITH QAYAPU RECIPIENTS ("PATIENTS")

INTERVIEW 2

Mr. Luis, 75 years old, originally from Ayacucho, works as a street vendor selling hats. He shared information based on his experiences with his children and grandchildren.

What is the qayapu for you?
"It is a healing practice performed for fright (susto) that children have."

Why did you seek the qayapu?
"For my grandchildren."

Do you think it is only for children or also for adults?
"For adults as well. For example, if I have an accident and get frightened, I cannot sleep and I need the qayapu."

What symptoms did they have that led you to seek the qayapu?
"They cry a lot, cannot sleep because of the fright, have no appetite—all of that happens until they are treated. Once the qayapu is done, the children return to normal."

Since when have you known about the practice of the qayapu?
"I have known it since I was young."

How do people feel after the qayapu?
"One feels lighter, more agile, because sometimes you feel heavy, not normal. It is like coming out of a shower—just like that."

What caught your attention most about the qayapu?
"Well, both women and men perform it. Some do it with genuine faith, while others do it quickly just to earn money. There are practitioners who truly know the practice; with them, a single session is enough for healing. Others do not perform it well, and children do not fully recover. But an experienced practitioner already knows well, so one qayapu is sufficient. Others say it must be done up to three times."

Did you have a good experience when you took your grandchildren?
"Yes, because they healed each time we took them."

How long does the qayapu last?
"It does not take more than five minutes—it is quick."

Do you think the qayapu is still important?
"Yes, it is important."

Do young people still practice the qayapu?
"No, nowadays some young people believe and others do not. We older people are the ones who continue practicing it. The traditional custom should continue."

INTERVIEW WITH QAYAPU RECIPIENTS ("PATIENTS")

INTERVIEW 3

Mrs. Dina, 57 years old, originally from Huamanga, sells cheese in the Central Market. She was very kind but provided brief responses, sharing her experience with the qayapu.

What is the qayapu for you?
"It is done when someone gets frightened. For example, when a baby falls, they start crying or get a fever. Since they fall, they get scared, so we take them to a man or to women who perform the qayapu. They pass an egg over them and pray, and the child improves."

What do they use when performing the qayapu?
"Some only pray, but others use an egg—that is more effective. Whenever they fall, I also pass any egg over them; if it is a farm egg, even better."

Since when have you known about the qayapu?
"Since I had my baby—when my child fell, I always took them to be treated. I am always attentive to that."

What changes do you notice after the qayapu?
"They become calm, they stop crying. Some who are vomiting stop as well."

Is the healing immediate or does it take several times?
"It is immediate. If it does not work, then I take them to the doctor."

Do you go first to the doctor or to the qayapu?
"First to the qayapu; if it does not help, then I take them to the doctor."

Do you think anyone can perform the qayapu?
"Yes, when someone prays, they can do it. But some make it effective immediately, while others just pray without meaning, and it does not help the child. Before, there was an elderly woman near the arch—I used to take my daughter to her. There used to be three elderly practitioners; now there is only one left at the arch."

Do you think the qayapu should continue to be practiced?
"Yes, of course."

INTERVIEW WITH QAYAPU RECIPIENTS ("PATIENTS")

INTERVIEW 4

Mrs. Ester, 61 years old, originally from Huamanga, lives in Santa Ana. She comes from a family of artisans and sells clothing for children and adults.

What is the qayapu for you?
"According to what my grandparents taught me, when babies get frightened their spirit leaves and wanders—it is no longer there. That is why they jump, vomit, do not want to eat, lie in bed, and their eyes look different—sunken, slightly turned. When you see a baby, their eyes show you they are frightened; they look different from when they are well."

Did you practice this with your children?
"Yes, I took my children since they were babies. When they got frightened after falling, they would jump like this (she gestures). I had to perform the qayapu. When you are young, you do not know, but later I did it myself—I would pass *rocoto* pepper with newspaper, crumple it like this and burn it, they told me. I also used *ají mirasol*, always passing it downward, never upward, all the way to the tip."

Where did you take your children for the qayapu?
"I took them here, by the market steps, to an elderly woman who sold flower water. There were also several elderly practitioners near Iglesia San Francisco de Asís (the arch). Now they are no longer there—I don't know what happened to them."

When they became ill, did you go first to the doctor or to the qayapu?
"No. My grandmother always advised me first—she told me in Quechua: 'Do not go first to the doctor; the *pacha* exists.' Based on that, I first performed the qayapu for my children, and only if it did not work would I go to the doctor. But the qayapu is effective. I learned since childhood because my grandmother, Edilberta Oriundo Pretell, practiced it. She also fermented *pichi*—she would place it in a *puyñu* (an oval jar with a wide opening), and with that she would pass it over the body while praying the Our Father. They also use eggs, candles lit in a church, *rocoto*, yellow chili, newspaper, carnations, roses—various things. The child's head is sucked gently using tap water with a bit of salt, from the head, hands, belly, down to the feet."

Is the healing immediate?
"No. After the qayapu, the baby or person falls asleep. When they sleep, it means they have received the healing. Then their appetite returns. It is done three or even four times. It is better on Tuesdays and Fridays—at six in the morning, noon, or six in the evening."

Does it need to be done in a quiet place, or does the location not matter?
"It does not matter. If it is done in the street, they should wear a little cap, but the times I performed it were at home."

Do you think the number of people seeking the qayapu has decreased?
"On the contrary, it has increased. Even adults now seek it—here, in Lima, everywhere. Since I also learned, people come to me and I perform it with an egg. When they ask me, I send them down there where the elderly practitioners are."

Do you recommend that the qayapu continue to be practiced?
"Of course. For example, in ancient times, how did the Incas heal everything—were there doctors or technology? I recommend that we learn from our ancestors and continue practicing it."

INTERVIEW WITH QAYAPU RECIPIENTS ("PATIENTS")

INTERVIEW 5

Mrs. Rosa, 53 years old, originally from Huamanga, has a stall in the Central Market where she sells various household items. She shared her experience with the qayapu.

Why did you seek the qayapu?
"I took my son when he was one and a half years old because he had fallen from the bed. He could not sleep, had vomiting, fever, and would not breastfeed—he was not the same."

Before taking him to the doctor, did you take him for the qayapu?
"Yes, because people told me it was fright (susto)."

Did he recover quickly?
"No, not immediately—it was gradual. I took him three times. They told me to bring him any day before noon."

Did they ask you to bring anything?
"Only prayers, a small carnation, and a cigarette."

Where did you go?
"To the arch, where the elderly practitioners are. I carried my baby, and he prayed over him."

What caught your attention the most?
"The calling back of his spirit."

How long does it take?
"About 10 to 15 minutes—it is not long."

Do you think medicine can cure this?
"No, because there is no medicine for fright. It can be confused with infection due to vomiting and diarrhea, but when it is fright, it does not go away."

Do you think the qayapu should continue to be practiced?
"Yes, of course. It will not disappear—it is always necessary. All children fall and need it."

Can anyone perform the qayapu?
"Those who have devotion—like a kind of power to call (the spirit). Not just anyone, because it does not work for everyone. They must have that belief."

Would you recommend that people continue practicing the qayapu?
"Yes, I would recommend it, but it depends on each parent. Some who do not believe say it is not true and take their children to the doctor. If they go without believing, I do not think it will work or be effective."

INTERVIEW WITH QAYAPU RECIPIENTS ("PATIENTS")

INTERVIEW 6

Mrs. María, 49 years old, originally from Ayacucho – Warpapicchu, shared her experience with the qayapu.

What experience have you had with the qayapu?
"I brought my daughter to have the qayapu done, and it was very good—it healed her when she was unwell. She was about eight years old when she had fallen and became frightened at night; she would jump like that, sometimes would not eat, and had diarrhea. But when they performed the qayapu, they did it well and she recovered."

What is the qayapu for you?
"It is something very good that helps heal your child. It is said that the little soul goes away somewhere, and they make it return."

What did they use for the qayapu?
"They passed an egg over her. They did it three times, in the mornings, close to noon."

What caught your attention about the qayapu?
"The way they prayed, called her by name, and sucked gently on her head."

Do you go first to the doctor or to the qayapu?
"No, I believe it is always the qayapu first, because the doctor does not know."

Do you think the number of people seeking the qayapu has increased?
"Yes, it has increased. I see many people coming to seek it—even adults get frightened. Children fall and become ill. I have always brought my children here for the qayapu."

Would you recommend that people continue practicing the qayapu?
"I would say each person decides for their children, but I like it because it heals my children."

Do you think anyone can perform the qayapu?
"I do not think so—it has to be someone who knows how. Not just anyone can do it without knowledge."

INTERVIEW WITH QAYAPU RECIPIENTS ("PATIENTS")

INTERVIEW 7

Mrs. Lourdes, 43 years old, originally from Ica, has lived in Ayacucho for more than 20 years. She shared her personal experience with the practice of the qayapu, a traditional ritual associated with treating fright (*susto*) in children.

Why did you seek the qayapu?
"For my older daughters. We used to come here to the market to work, and they would fall. People would tell me they had the 'evil eye,' and they would direct me to a woman in the market who sells condiments—she is the one who performs the qayapu."

What symptoms did they have?
"They could not sleep, their eyes stayed half open, and at night they cried. When it was colic, I gave them *Gaseovet*, but it did not help. I would rub their forehead with salted water, but it still did not go away."

How many times did you take them?
"Three times—on Tuesdays and Thursdays before six in the evening. With my older daughters, yes; with my youngest, no, because she does not get frightened anymore."

Did you take them first to the doctor or to the qayapu?
"No, always first to the qayapu. If it does not work and they continue with diarrhea, then I take them to the doctor. After the first session, they cry less; after the second and third, they improve."

How long does it take?
"Only ten to fifteen minutes—it is not long."

What did they ask you to bring?
"A small shirt, because during the prayer they sweat since they resist being touched. They did not ask for candles, coconut, or cigarettes—only prayer."

Do you think the number of people seeking the qayapu has decreased?
"Yes, I think it has decreased, because the older practitioners no longer come out. I could no longer find the woman who treated my daughters."

Do you think anyone can perform the qayapu?
"I do not think so."

Would you recommend that people continue practicing the qayapu?
"Yes, I would recommend it. But for us older generations, we had those beliefs. Nowadays, young people rely more on going first to a doctor, consulting a specialist about what is happening. In our time, as first-time parents and with little guidance, we went first to the qayapu—especially if the child had fallen or had stomach problems. Now I also recommend seeking a specialist, since professionals have more patience and know how to treat these conditions. It could be complemented with the qayapu."

INTERVIEW WITH QAYAPU RECIPIENTS ("PATIENTS")

INTERVIEW 8

Mrs. María, 58 years old, originally from Ocros, has lived in Ayacucho for many years and sells goods in the market. She did not agree to be recorded, so her responses were very brief.

What is the qayapu for you?
"It is the calling that is done for fright. When a child gets frightened, they become ill—they do not want to eat, their stomach and head hurt, and their eyes become sunken."

Why do they become frightened?
"For example, a rooster comes and kicks the child, and they get frightened. When that happens, they are taken for the qayapu."

What did they ask you to bring for the qayapu?
"They did not ask me for anything—only prayer. When the calling is finished, you pay what you wish. I only took my child once, and the next day he was already better and slept well. I took him to the elderly man at Iglesia San Francisco de Asís—only once."

INTERVIEW WITH QAYAPU RECIPIENTS ("PATIENTS")

INTERVIEW 9

Mrs. Cecilia, 30 years old, originally from Huancayo, has been living in Ayacucho for four years.

What has been your experience with the qayapu?
"I come from Huancayo, and there most people believe in it. I had nephews who used to get frightened, but I did not believe in it until I had my daughter. When she was almost one month old, she would stick out her tongue in the evenings. Then someone told us it was 'mipa de gato,' so we took her to a woman who passed a kitten over her, and she stopped doing it. The woman told me that my baby had been frightened in the womb because I do not like cats. I took her twice."

When they performed the qayapu, what did they ask you to bring?
"They asked me to dress her in white, and to bring alum, a small candle, coca leaves, a cigarette, and anise. The last time, when my husband's grandmother passed away, her spirit frightened my daughter, and she kept closing one eye repeatedly. We took her again, and the woman asked for white roses. She passed them over her three times, and it went away."

Was it done in a public place?
"No, it was at her house. The three of us went—my husband, my daughter, and I."

How quickly did you notice improvement?
"Almost immediately. She asked me to come at noon, and by the afternoon my daughter had stopped doing that."

What is the qayapu for you?
“As I mentioned, in Huancayo my grandparents and my mother explained that the qayapu is mainly when a spirit, an entity, our ancestors, or some presence takes hold of the baby. Since babies are pure souls, not with bad intentions, but just by being seen or approached, many things can happen to them. That is why they get frightened, and the qayapu is performed to call the soul back so that health returns.”

Do you think the qayapu should continue to be practiced?
“From my experience, I did not believe in it at first. When I was young, my nephews had it and I did not take it seriously. But when I became a mother, I began to believe, because it happened more than once. So yes, it should continue to be practiced. When I looked for it here in Ayacucho, people did not know what it was—I had to search, and older people were the ones who told me where to find it.”

Where did you take your child?
“To the Arco de 28 de Julio, to a woman there.”

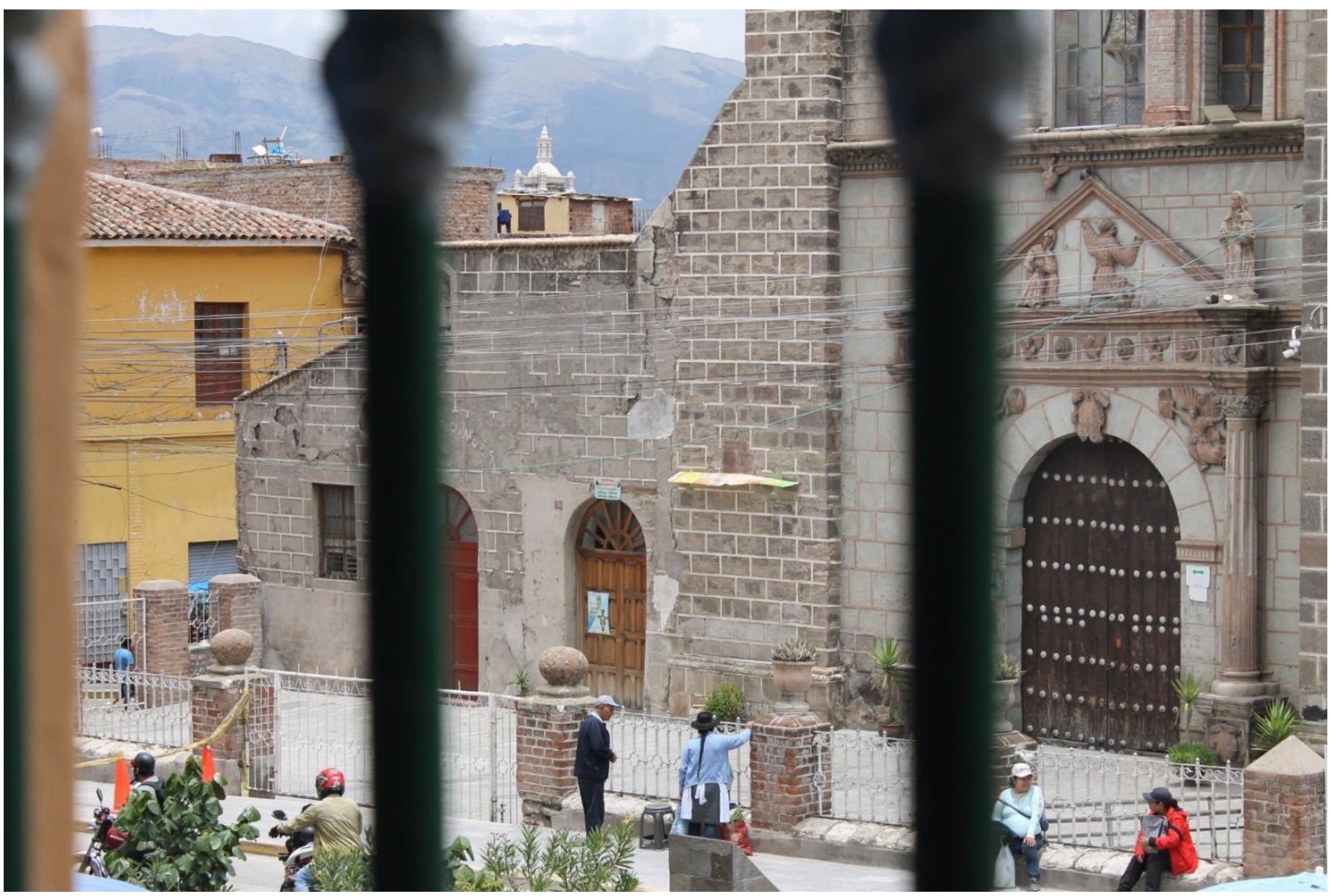

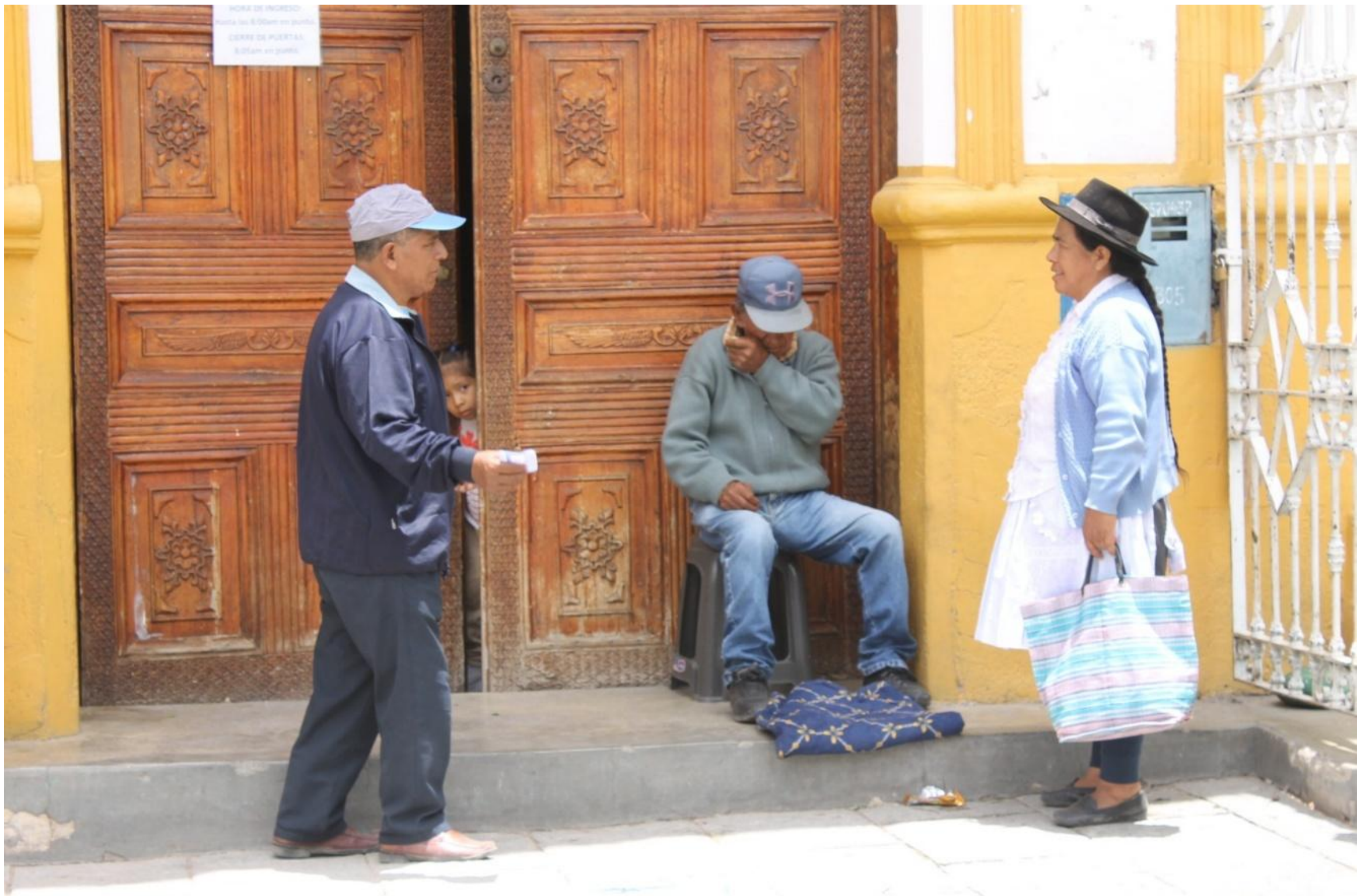

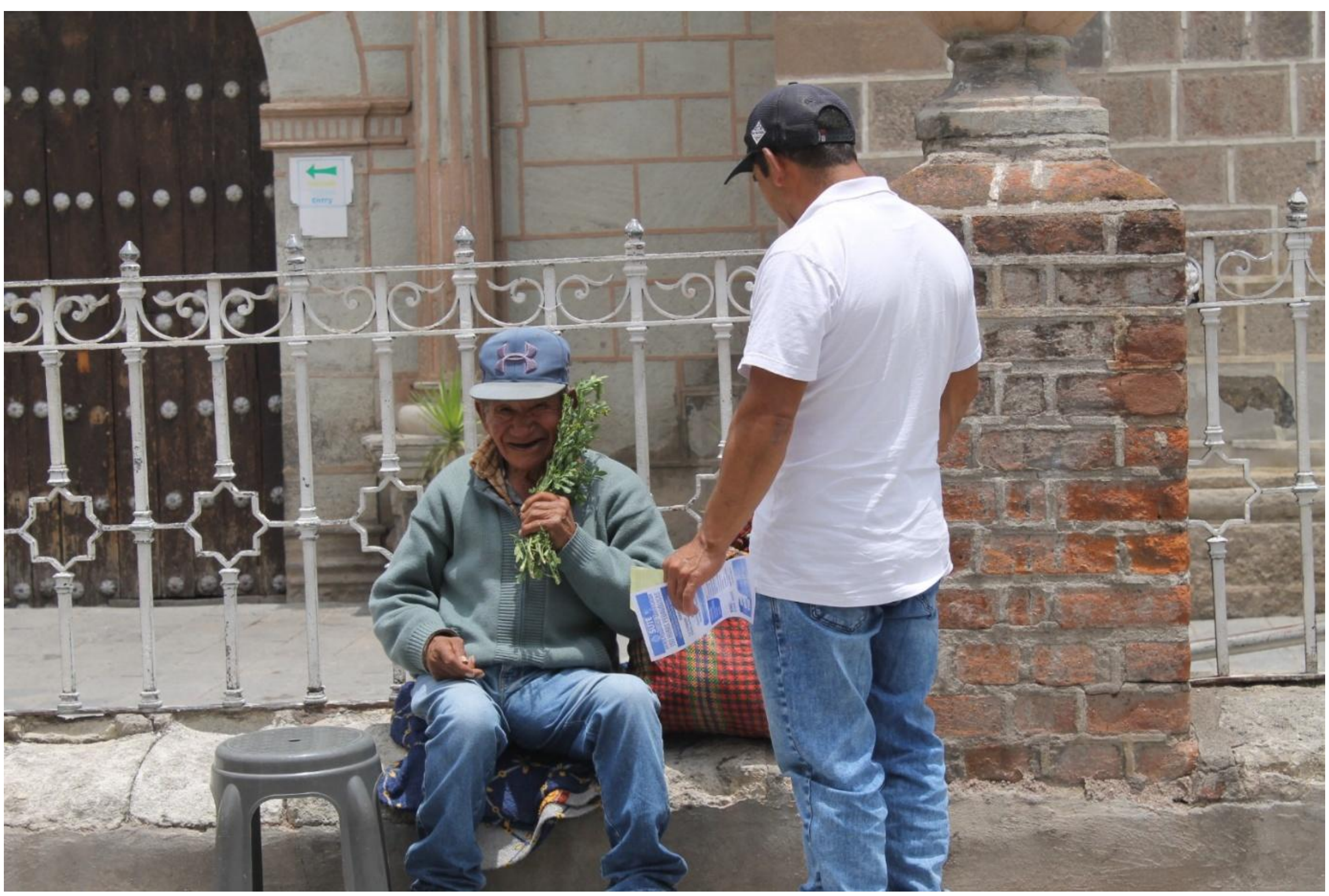

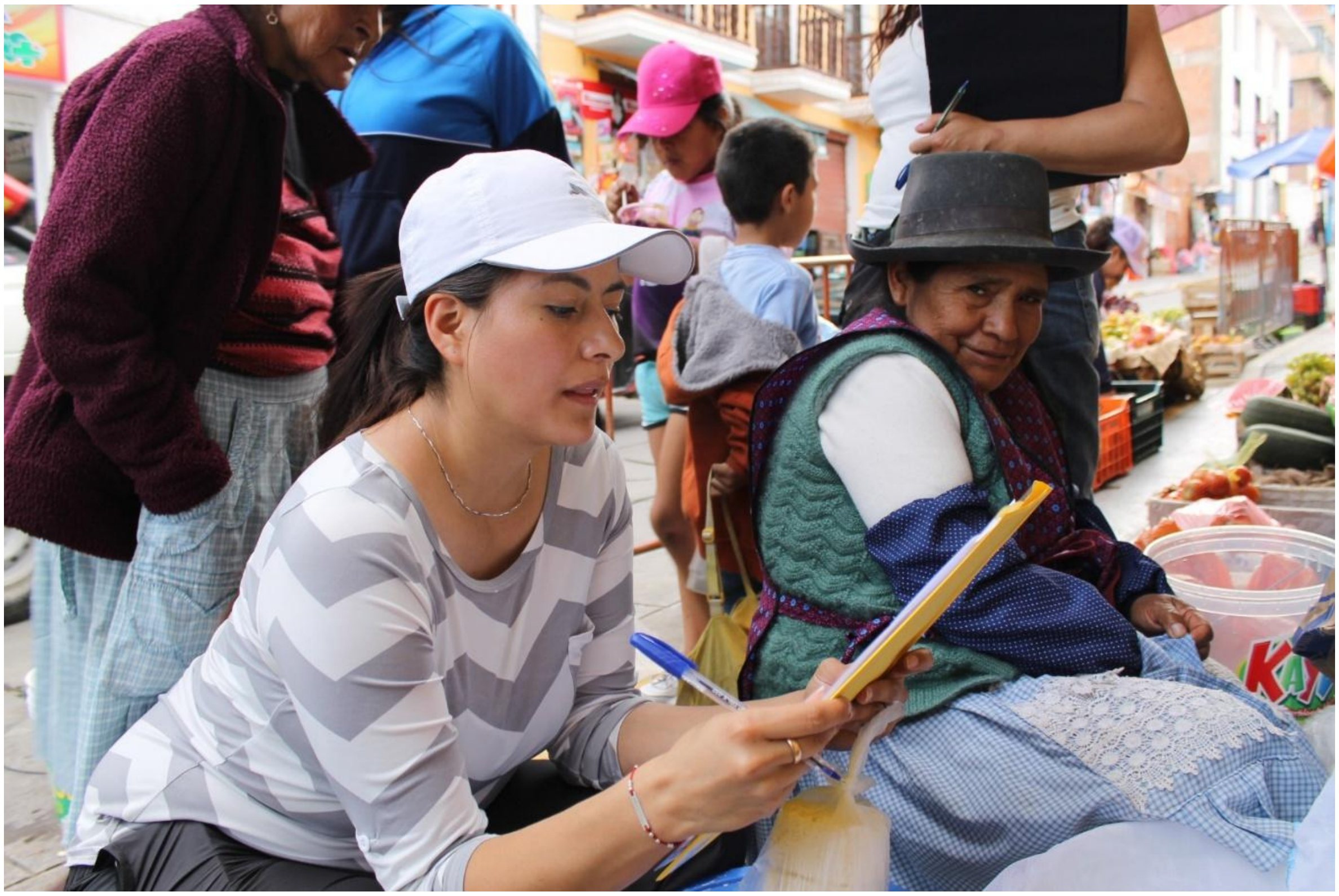

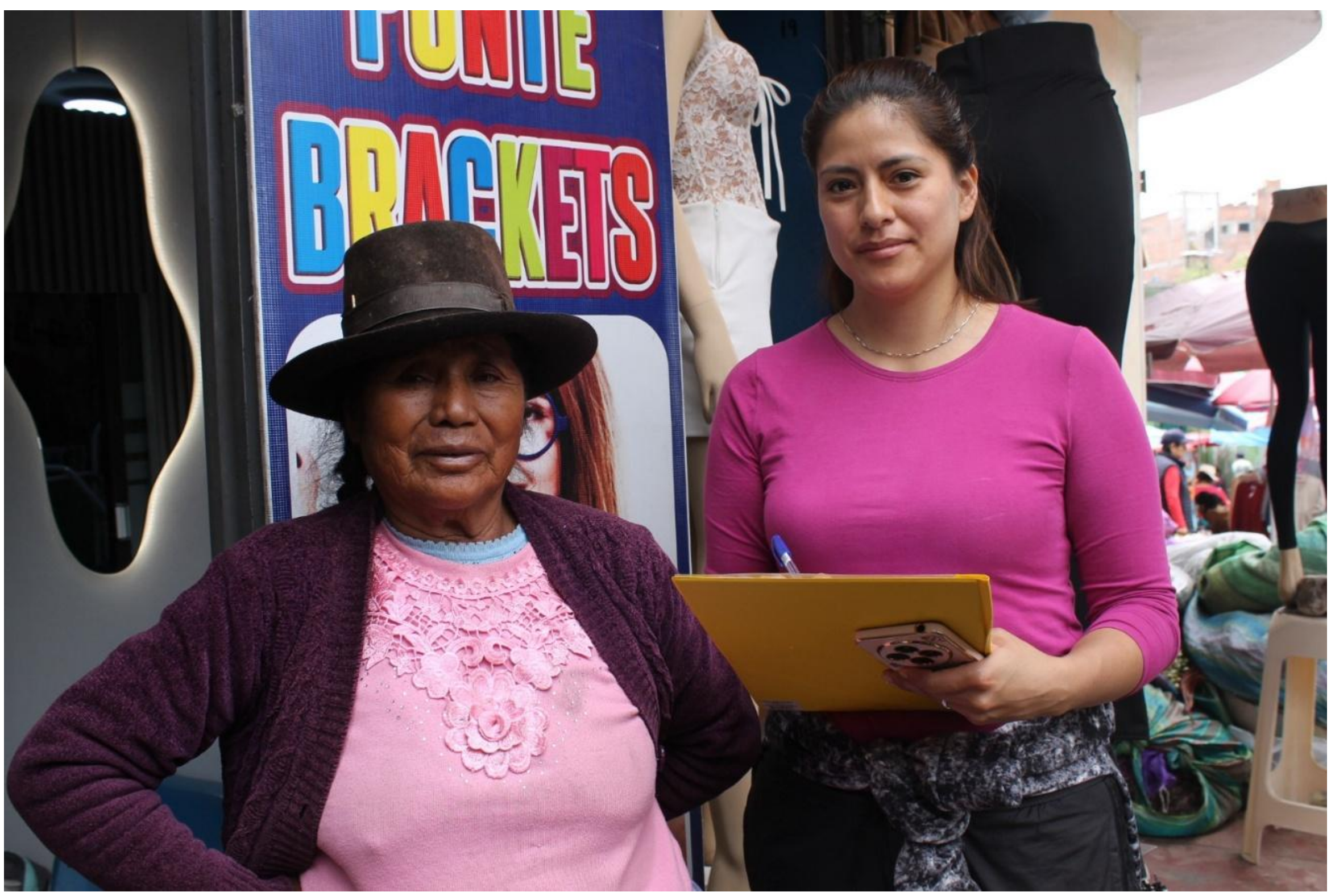
BRACKETS

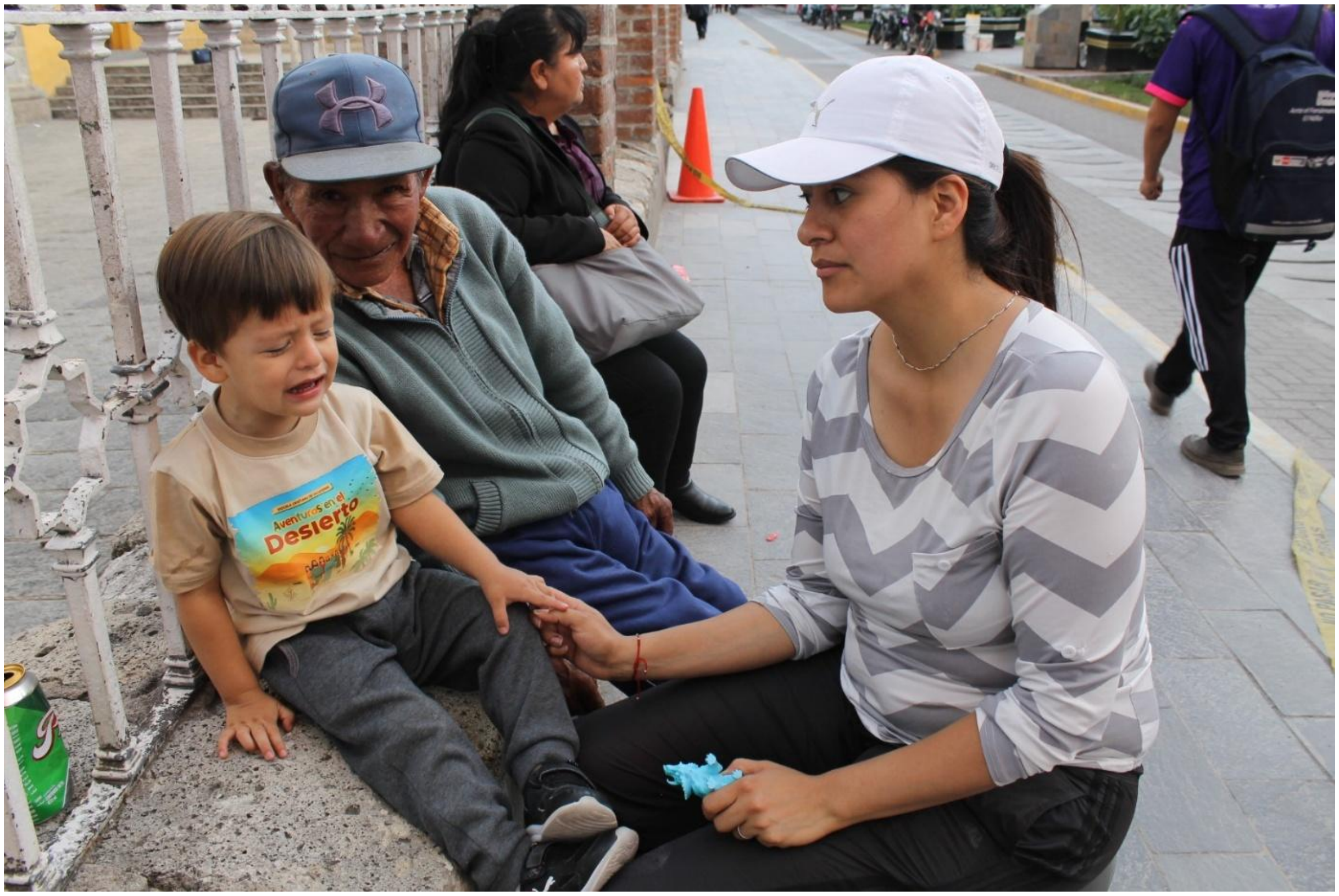

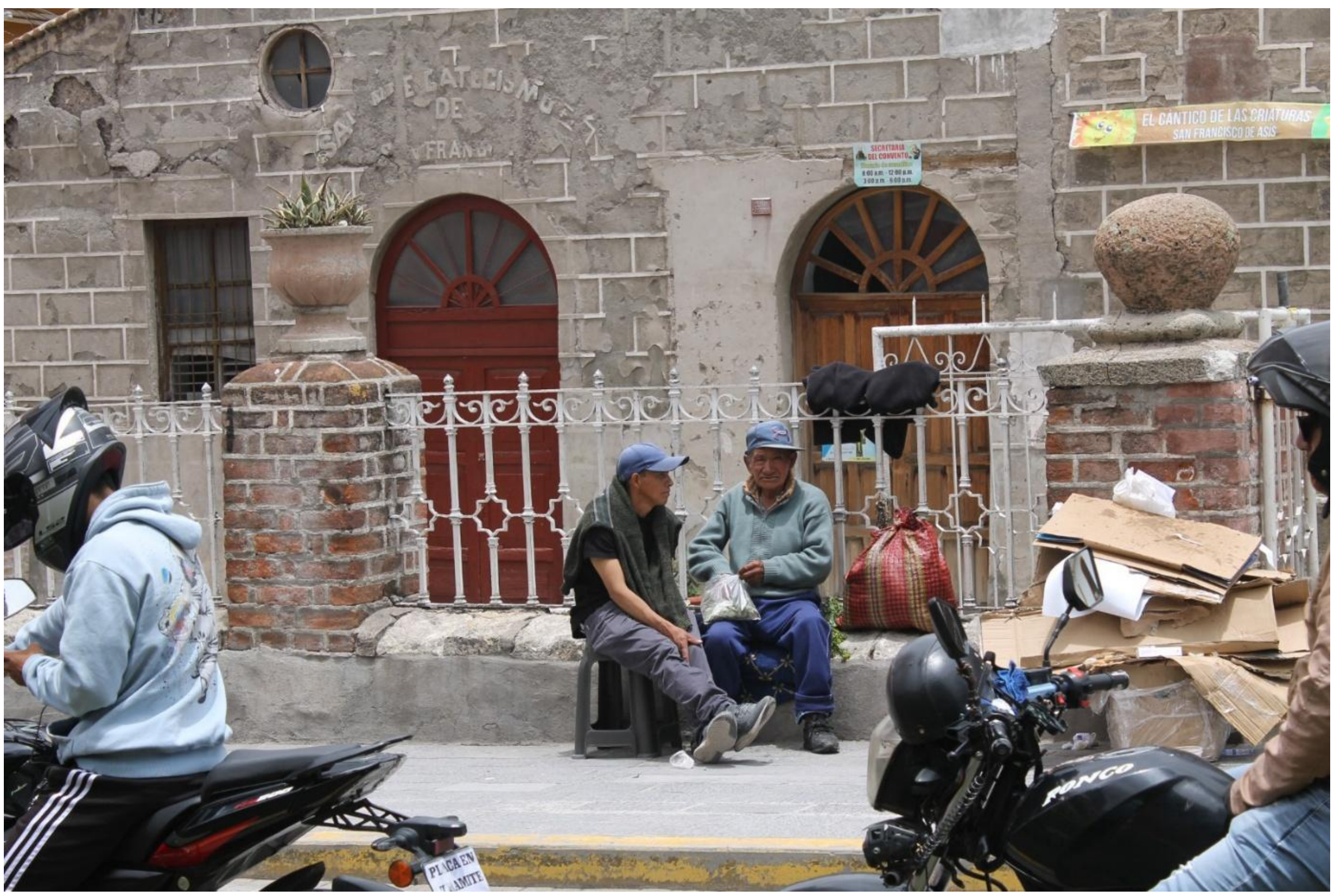

Some recordings:

https://drive.google.com/file/d/1ChhRh55QkNBc9jKavRbBhBvMWRHECzvS/view?usp=drive_link

https://drive.google.com/file/d/1nNo5fiyCadFUyIahVwSR83zVqxNkfqhP/view?usp=drive_link

https://drive.google.com/file/d/1GmVvwSjBCwmPdznnRsMPDf0La7mfCY96/view?usp=drive_link

https://drive.google.com/file/d/1ajU3HPc0f8PDt3C-WtlqtBiWQMHkVeP2/view

https://drive.google.com/file/d/1ZOcOFILba4H4pjeyxbnYmfOi0oq5ExsC/view?usp=drive_link

https://drive.google.com/file/d/1iNjHalg7khzgyJyfxcFTkBcCq5IWBhya/view?usp=drive_link (ritual of photography)

https://drive.google.com/file/d/1GAxRuQMDCJUtyeoXipUBogWUNDWEotUb/view?usp=drive_link

https://drive.google.com/file/d/1kn0nr6JEdiHlK3e7ANLMrWRP1U6PEndV/view?usp=drive_link

https://drive.google.com/file/d/1pldHe_lDUdXicC4Maekh6WGttnNdb3jG/view?usp=drive_link

https://drive.google.com/file/d/1xDpPWn8WdbVRhcUNTGwnwytNWGOSTZ7A/view?usp=drive_link

https://drive.google.com/file/d/1ILD9uyX9qnkXjrxF_jxRAckLdrtdMIQk/view?usp=drive_link

https://drive.google.com/file/d/1itkV1c4_nH0kUQFSI6b2eULiBLnZ1LOR/view?usp=drive_link

https://drive.google.com/file/d/18cPQFYlWY9wNBeh83TLxiVbK_q3uXgS8/view?usp=drive_link

https://drive.google.com/file/d/14Z3TaON0cnQvHi69u1Xpfq7Fgm6nC-3E/view?usp=drive_link

https://drive.google.com/drive/folders/1Gh-dc6sgtkIupLA2bToAH34ZrR59zLMS?usp=drive_link

https://drive.google.com/drive/folders/1RKF-5NlgzNjmlJCNUtXEkhk3-MIfi7Eu?usp=drive_link
